# Dual transcriptomic profiling of *Staphylococcus aureus* endocarditis in a porcine model reveals strong parallels with human infection

**DOI:** 10.1128/mbio.02316-25

**Published:** 2025-10-31

**Authors:** Begoña García, Aritza Conty, Amaya Fernández-Celis, Daniel Mouzo, Carmen Gil, Nahiara Garmendia-Antoñana, Ana Navascues, Carmen Ezpeleta, Cristina Solano, Ivan Pasquier, Virginia Álvarez, Rafael Sádaba, David Gómez, Natalia López-Andres, Iñigo Lasa

**Affiliations:** 1 Laboratory of Microbial Pathogenesis, Navarrabiomed-Hospital Universitario de Navarra (HUN)-Universidad Pública de Navarra (UPNA), IDISNA 540940 , Pamplona, Navarre, Spain; 2 Translational Cardiology Laboratory, Navarrabiomed-Hospital Universitario de Navarra (HUN)-Universidad Pública de Navarra (UPNA), IDISNA 540940 , Pamplona, Navarre, Spain; 3 Unit of Translational Bioinformatics, Navarrabiomed-Hospital Universitario de Navarra (HUN)-Universidad Pública de Navarra (UPNA), IDISNA 540940 , Pamplona, Navarre, Spain; 4 Department of Clinical Microbiology, Hospital Universitario de Navarra (HUN), Pamplona, Navarre, Spain; 5 Biological and Environmental Science and Engineering Division, King Abdullah University of Science and Technology (KAUST) 127355, Thuwal, Saudi Arabia; The University of Tennessee Knoxville, Knoxville, Tennessee, USA

**Keywords:** endocarditis, dual RNA-seq, *Staphylococcus aureus*, human heart valves

## Abstract

Understanding the intricate interactions between *Staphylococcus aureus* and valve cells during the course of endocarditis is crucial for developing more effective treatments for an infection that has a mortality rate exceeding 30%. To investigate the molecular adaptations occurring in both the valve tissue and bacteria during infection, we performed dual RNA-seq analysis on valves from patients undergoing valve replacement surgery due to *S. aureus* endocarditis as well as from an experimental pig model of the disease. The results revealed remarkably consistent expression profiles between human and pig samples, characterized by endothelial damage and the activation of valve interstitial cells, leading to excessive inflammation, heightened coagulation cascades, collagen degradation, and extracellular matrix remodeling. Additionally, transcriptional profiling of *S. aureus* on infected valves showed the upregulation of genes involved in iron acquisition, capsule synthesis, protease production, and adhesin formation, along with downregulation of genes associated with pyrimidine metabolism and nitrate reduction. These findings provide a detailed map of the transcriptional landscape defining both the infected valve tissue and *S. aureus* during active endocarditis.

**IMPORTANCE:**

To gain a full understanding of how bacteria infect tissues, it is necessary to characterize both the bacteria and the tissue at the time of infection. However, this analysis is very complex because it involves obtaining infected tissue directly from patients and analyzing it with minimal processing to preserve the characteristics of the natural state of infection. In this study, we examined the gene expression profiles of replaced heart valves from patients with *Staphylococcus aureus* infection and compared them with pig valves experimentally infected with the same bacteria. Our findings provide a detailed insight into the changes occurring in the infected tissue and the bacterial adaptations required for multiplication and survival on the valve tissue. Notably, the strong similarity observed between human and porcine valves confirms that the porcine endocarditis model closely mirrors the human condition, making it a valuable tool for testing new therapies against this serious infection.

## INTRODUCTION

Infective endocarditis (IE) is characterized by microbial colonization and persistence on the endothelium of heart valves. While IE can develop on healthy valves, preexisting conditions such as rheumatic heart disease, degenerative valve disease, or anatomical abnormalities increase the vulnerability of the endothelial surface to bacterial adhesion, thus facilitating infection (1–4). Despite being relatively uncommon, with an incidence of 3–10 cases per 100,000 individuals per year, IE is associated with severe complications and a mortality rate exceeding 30% (5–9).

*Staphylococcus aureus* is the leading cause of native valve IE, accounting for 27% of all IE cases (10–13). The development of IE depends on a strong interaction between *S. aureus* and inflamed or damaged endocardial tissue, which prevents the bacteria from being carried away in the bloodstream. *S. aureus* expresses multiple adhesins, including clumping factors A and B (ClfA and ClfB), fibronectin-binding protein A (FnBPA), protein A (Spa), and von Willebrand adhesin (14–20), which enable the bacterium to adhere directly to fibrin, collagen, fibronectin, and von Willebrand factor present in the damaged endothelium. Upon adhesion, *S. aureus* components are detected by pattern recognition receptors on endothelial cells, platelets, and leukocytes, triggering the release of cytokines. This response activates endothelial cells and monocytes, prompting the release of thromboplastin and initiating the coagulation cascade. The resulting thrombin production activates platelets and increases fibrin production, further promoting bacterial adhesion and contributing to the development of thrombotic vegetations composed of fibrin, platelets, leukocytes, host proteins, and bacteria (21–23). The formation of these vegetations usually results in valve destruction, sepsis, or embolization, all of which carry high mortality rates (24–26).

To survive and proliferate within the valve endothelium, *S. aureus* must adjust its gene expression to suit this specific environment (27). Simultaneously, endothelial cells must modulate their gene expression to mitigate bacterial damage and activate defense mechanisms. A method used to examine the transcriptional profiles of both bacteria and host tissue during infection is dual RNA sequencing (28–30). This technique involves purifying total RNA from an infected tissue and simultaneously quantifying bacterial and host RNA transcripts. In the context of endocarditis, and due to the challenges of obtaining valve-infected tissue, dual RNA sequencing has been employed to study the transcriptomic profiles of *S. aureus*-infected macrophages or human umbilical vein endothelial cell lines (31, 32). While these studies provide valuable insights, they do not fully replicate the complexity of the *in vivo* infection process, as they lack factors such as blood flow, cellular heterogeneity, and the presence of the immune system, all of which play crucial roles in the progression of infection.

In this study, we employed a dual RNA-seq approach to explore the interactions between *S. aureus* and cardiac valve cells, aiming to identify a distinct transcriptional profile associated with this pathology. Initially, this technique was used to analyze infected valves from pigs, where the endocarditis process was experimentally induced. Then, it was applied to valves from patients who had undergone valve replacement surgery due to acute endocarditis. Remarkably, the results show an enormous similarity in the transcriptional changes that occur between human and porcine valves, demonstrating that the simultaneous RNA sequencing technique provides an irreplaceable insight into what happens during the pathogen-host interaction in infected tissue. The results showed that valve infection leads to hyperactivation of interstitial cells, accompanied by excessive inflammation, coagulation, and remodeling of the extracellular matrix. From the perspective of *S. aureus*, results revealed that the bacterium activates multiple virulence mechanisms, including iron acquisition systems, capsule formation, production of proteases and hemolysins, and expression of adhesins. The consistency of results between porcine and human valves demonstrates the existence of a global transcriptional profile characteristic of the acute endocarditis process and confirms that the porcine model is an important resource for testing patient treatment regimens and identifying new therapeutic targets for the treatment of infection.

## RESULTS

### Histological and molecular signature in valves from *S. aureus* endocarditis

**Fig 1:**
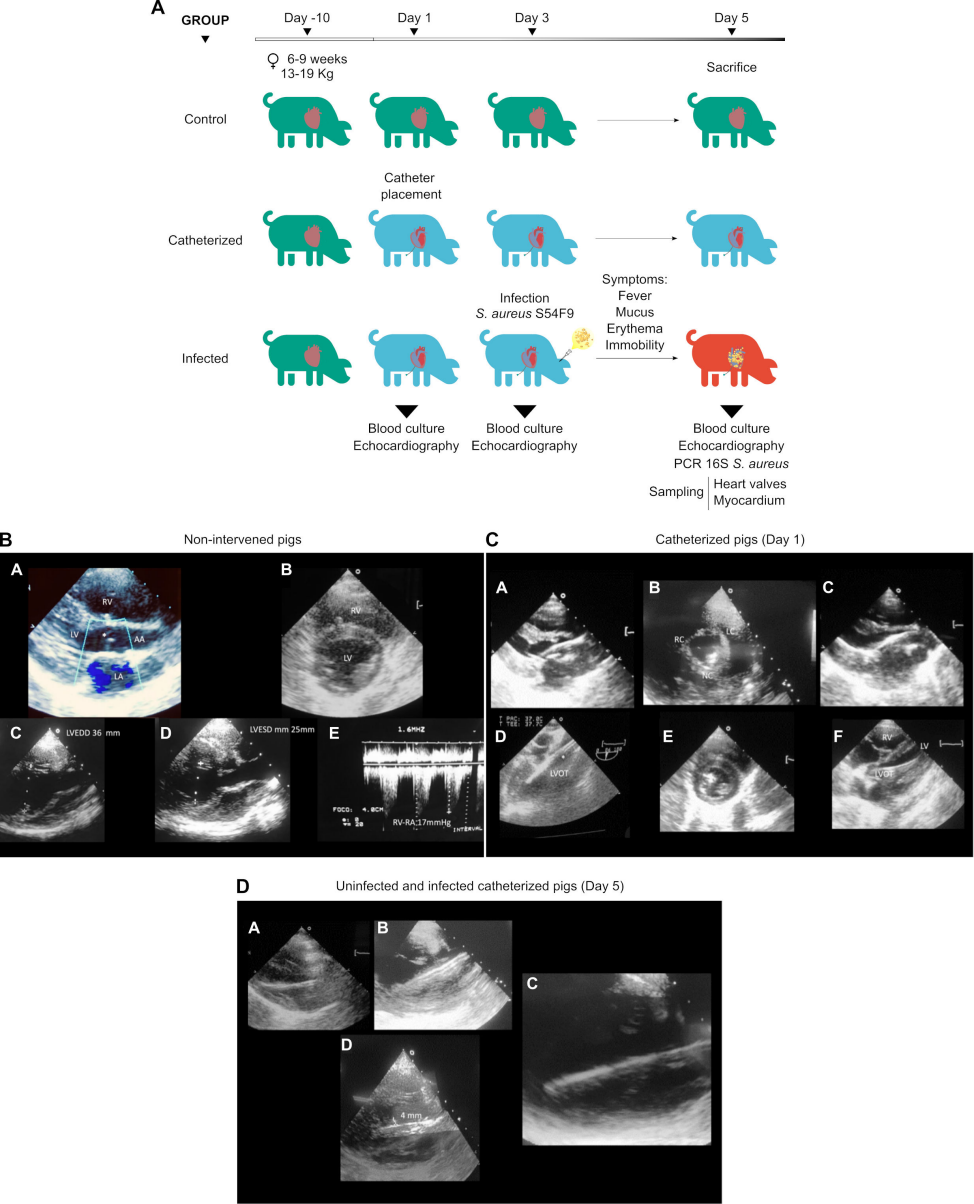
Porcine *S. aureus* IE model and echocardiographic evaluation. (**A**) Experimental design of the porcine *S. aureus* IE model. Three groups of pigs (*n* = 6 per group) were used: healthy controls (Control), catheterized but uninfected animals (Catheterized), and catheterized animals infected with *S. aureus* S54F9 (Infected). Following a 10-day stabilization period, catheterization was performed on day 1, infection on day 3, and euthanasia on day 5 after clinical signs appeared in the infected group. In the infected group, blood samples were collected for culture, and transthoracic echocardiography (TTE) was performed on days 1, 3, and 5. Transesophageal echocardiography (TEE) was also performed on day 5. In the catheterized group, blood cultures and TTE were performed on days 1 and 5. In the Control group, blood samples were taken for culture on day 5 only. After euthanasia, valve tissues were harvested for further analysis, including PCR detection of *S. aureus* 16S rRNA to confirm infection status. Results from blood cultures and PCRs are provided in Table S1. (**B**) Transthoracic echocardiographic images from pigs of catheterized and infected groups taken on day 1, before catheter placement (non-intervened pigs). (A) Parasternal long-axis view (PLAX). AA (ascending aorta); LA (left appendage); LV (left ventricle); RV (right ventricle); *aortic valve. (B) Short axis view: LV (left ventricle); RV (right ventricle). (C and D) LVEDD and LVESD (left ventricle end diastolic diameter and end systolic diameter). Ejection fraction by Teichholz method: 59%. (E) Right ventricle-right atrium pressure gradient: 17 mmHg, Systolic pulmonary artery pressure estimation around 23–27 mmHg. (**C**) TTE and TEE images taken on day 1 from catheterized pigs after catheter placement. (A and C) TTE PLAX views showing the catheter passing through the aortic valve into the left ventricle, with different orientations. (B) TTE short-axis view showing the three aortic cusps (LC: left coronary, RC: right coronary, NC: non-coronary) and the catheter passing through the non-coronary leaflet. (D) TEE three-chamber view with the aortic annulus (*) and catheter extending into the anterior mitral leaflet. (E) TTE short-axis view, with the catheter appearing as a hyperechogenic signal. (F) TTE five-chamber view showing the catheter in the left ventricular outflow tract (LVOT) entering the left ventricle. (**D**) Day 5 echocardiographic images from pigs of the catheterized and infected groups. (A–C) TTE PLAX views showing catheter-associated thickening several days after implantation, observed in both uninfected and infected animals. (D) TTE image from an infected pig showing a 4 mm nodular lesion attached to the catheter, consistent with vegetation formation.

**Fig 2:**
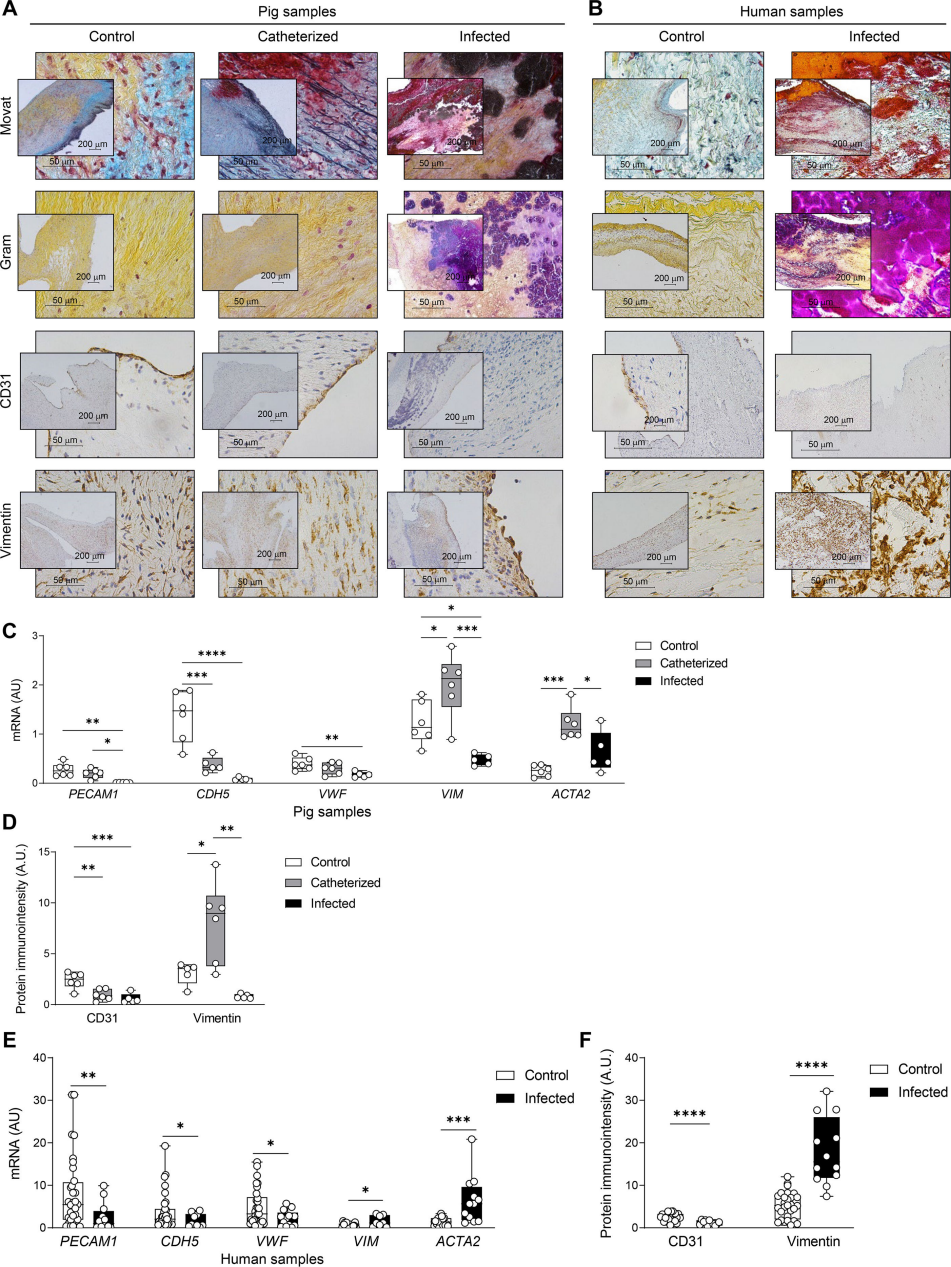
Histological and molecular signatures in valves from *S. aureus*-associated endocarditis. (**A**) Representative valve sections from control, catheterized, and infected pigs and (**B**) valves from controls and patients with IE caused by *S. aureus,* stained with Movat’s pentachromic and Gram, and immunostained for CD31 and vimentin. (**C**) mRNA expression levels of *PECAM-1, CDH5, VWF, VIM,* and *ACTA2* in cardiac valves from the control (*n* = 6), catheterized (*n* = 6, *CDH5 n* = 5), and infected pigs (*n* = 5). (**D**) Quantification of CD31 and vimentin immunostaining in porcine valves across all three experimental groups. (**E**) mRNA levels of *PECAM-1, CDH5, VWF, VIM,* and *ACTA2* in cardiac valves from control (*PECAM-1 n* = 32, *CDH5 n* = 31, *VWF n* = 30, *VIM n* = 17, and *ACTA2 n* = 19) and IE patients (*PECAM-1 n* = 12, *CDH5 n* = 10, *VWF n* = 13, *VIM n* = 8, and *ACTA2 n* = 13). (**F**) Quantification of CD31 and vimentin immunostaining in human cardiac valves from control and IE patient groups. For qPCR analyses, mRNA levels (expressed in arbitrary units [A.U.]) were normalized to *18S, HPRT, ACTB,* and *GAPDH* levels. Boxplots display the interquartile range (25th and 75th percentiles) with the median indicated by the center line. Whiskers show maximum and minimum values. *P < 0.05, **P < 0.01, ***P < 0.001, ****P < 0.0001.

To enhance our understanding of the interaction between *S. aureus* and heart valve tissue during endocarditis, we adapted an animal model of IE in pigs (33). A valvular lesion was induced by inserting a catheter through the carotid artery into the left ventricle of pigs. Three days after catheter implantation, the infected group received a single intravenous dose of *S. aureus* S54F9, a strain previously isolated from pigs and employed in porcine infection models (34, 35). After euthanasia, cardiac valves from three groups were collected for histological analysis and RNA purification: the control group (neither catheterized nor infected), the catheterized group (catheterized and not infected), and the infected group (both catheterized and infected) (Fig. 1; Table S1). In cardiac valves of infected pigs, Gram and Movat pentachrome staining confirmed the presence of vegetations composed of Gram-positive cocci and disruption of the three-layer histological structure, abnormal fibrin distribution, disorganization of the extracellular matrix with disrupted elastic fibers and collagen networks, and a significant reduction in proteoglycan content (Fig. 2A). Endothelial loss was evidenced by CD31 immunostaining in infected animals (Fig. 2A). This observation correlated with decreased mRNA levels of the endothelial markers *PECAM-1*, *CDH5,* and *VWF* in the catheterized animals that was more pronounced in the infected group (Fig. 2C), and a decrease in CD31 immunostaining in infected animals (Fig. 2A and D). Additionally, quantification of the valvular interstitial cell (VIC) marker vimentin revealed increased levels in catheterized animals, whereas in the infected group, vimentin expression was decreased and confined to the “healthy” areas of the valves (Fig. 2A and D). Consistently, mRNA levels of the VIC marker *VIM* were significantly higher in catheterized animals and lower in infected animals compared to controls, whereas those of the VIC activation marker *ACTA2* showed an upward trend in infected animals compared to controls, though this increase was not statistically significant (*P* = 0.06) (Fig. 2C).

To determine whether the histopathological features observed in the experimental swine model accurately reflected what occurs during human endocarditis, we analyzed the histological profile of valves from patients who underwent replacement surgery for *S. aureus*-induced IE. Fourteen IE valves obtained during cardiac surgery were analyzed using semi-quantitative methods, and the results were compared with data from 34 control valves obtained through autopsies (36). The mean age of the patients with IE was 56 ± 11 years, being 43% male. Comorbidities were present in 71% of the patients, with arterial hypertension in 29%, diabetes mellitus in 29%, cerebrovascular disease in 29%, obesity in 57%, and coronary artery disease in 14%. The most common presentation was acute in 86% episodes (subacute in 14%). At hospital admission, the primary diagnosis was “fever of unknown origin” (85%), and 15% of patients presented with septic shock. Inflammatory markers were markedly elevated in the initial analysis (C-reactive protein 213 mg/L; normal value <5 mg/L). Blood cultures were positive for *S. aureus* in all patients, and PCR analysis of excised valve tissue also confirmed the presence of *S. aureus*. Vegetations were identified in all cases, with valve perforation observed in 71% and perivalvular abscesses confirmed in 43%. The bacterial load in these samples was completely unknown, as the patients had undergone intensive antibiotic treatment prior to surgery. The mean duration of intravenous antibiotic therapy prior to surgery was 6.6 ± 3 days. As observed in the porcine model, Gram and Movat pentachrome staining in human IE samples confirmed the presence of vegetations, tissue disorganization, endothelial layer disruption, as well as the VIC activation profile (Fig. 2B). Accordingly, molecular analysis revealed a decrease in endothelial markers *PECAM-1*, *CDH5,* and *VWF* in the infected group accompanied by an increase in VIC activation markers *VIM* and *ACTA2* (Fig. 2E). Quantification of CD31 and vimentin immunostaining in cardiac valves from controls and infected valves followed the same trend (Fig. 2F). Taken together, these results reveal similar histological changes in valves from the pig infection model and those from patients undergoing valve replacement surgery.

### Transcriptional signature of the pig heart valve in *S. aureus*-associated endocarditis

To simultaneously profile the transcriptional activity of infected valve cells and *S. aureus*, total RNA was extracted from pig heart valves. For the control and catheterized groups, three samples each (*n* = 3 per group) were selected based on higher RNA yield and integrity (RIN > 7 or equivalent bioanalyzer profile). For the infected group, samples with detectable bacterial ribosomal RNA (16S/23S) and bioanalyzer profiles indicating adequate RNA quality were prioritized (*n* = 4) (Table S2). cDNA libraries were prepared and sequenced using the Illumina NextSeq 500 platform (Vertis Biotechnologie AG). Each sample yielded between 27.3 and 44.9 million reads (Table S3). In the infected group, an average of 40.8% of reads aligned to the *Sus scrofa* reference genome (GCA_000003025), while 58.6% aligned to the *S. aureus* S54F9 genome (GCA_001281605). A substantial proportion of reads in both genomes mapped to coding sequences (CDS), providing sufficient coverage for subsequent differential expression analysis (DEA).

**Fig 3:**
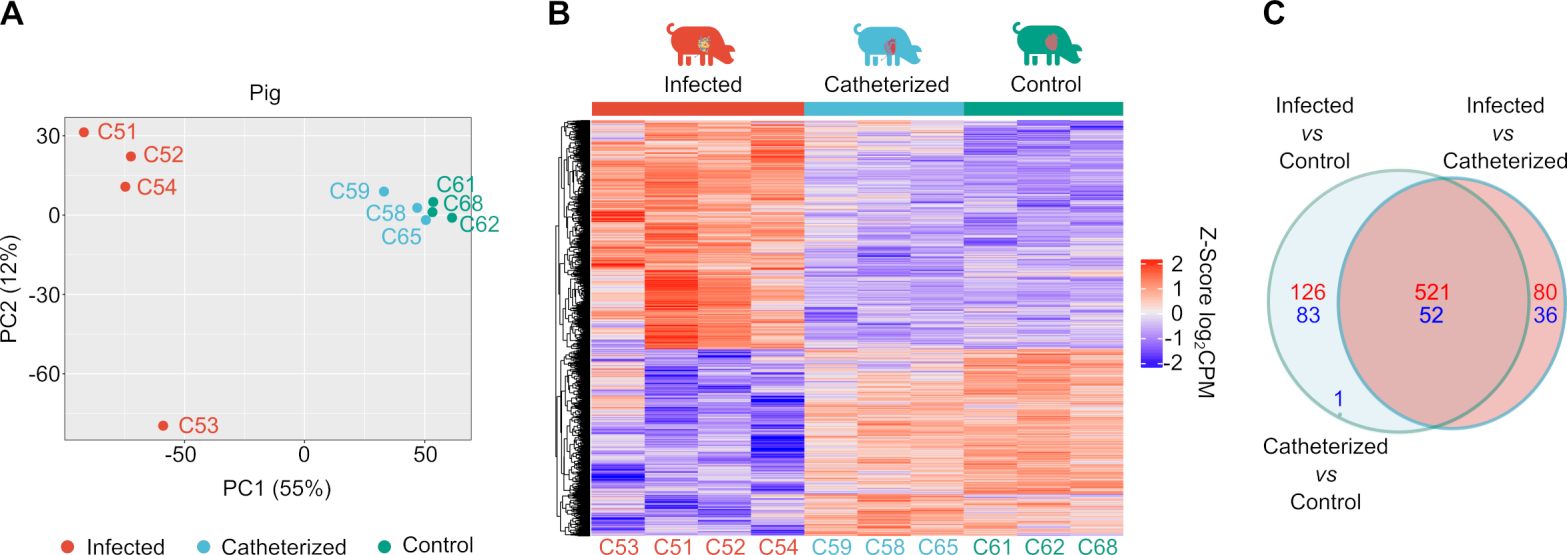
Analysis of differentially expressed genes in the valve tissue of pigs during *in vivo* infection. (**A**) PCA plot of porcine heart valves. The samples are grouped into three categories based on their expression profiles, corresponding to each study group: control group, catheterized group, and infected group. The animal numbers for the respective samples are indicated. (**B**) Analysis of differentially expressed genes in valve tissues of pigs within the experimental groups (control, catheterized, and infected). The heat maps illustrate the expression profile of differentially expressed genes in each of the specified experimental groups. Genes with a *P*-value adjustment <0.05 are depicted. Colors represent z-scores of voom-transformed log₂-counts-per-million (logCPM) values. Raw counts were normalized using TMM and transformed with voom; each gene’s expression was then centered and scaled across samples, so the color key (–2 to + 2) indicates standard deviations from the mean expression of that gene. The number of animals in each sample is also indicated. (**C**) A Venn diagram illustrates the number of differentially expressed genes (with a *P*-value adjustment <0.05 and a log_2_ FC >2/<−2) in the three indicated comparisons, with an overlay showing the number of commonly regulated genes. In the catheterized vs control comparison, only one gene was differentially expressed (downregulated). This gene was also found to be downregulated in the infected vs control comparison.

Gene expression patterns within each group of valves were first assessed using principal component analysis (PCA) (Fig. 3A). A heat map of the gene expression profiles showed that infected tissues had a significantly altered expression signature compared to both healthy and catheter-injured tissues (Fig. 3B). In contrast, the healthy and catheter-injured tissues were highly similar in their gene expression profiles. Compared to healthy and catheter-injured valves, the infected valves showed significant transcriptional changes, with 647 and 601 genes significantly upregulated, and 135 and 88 genes significantly downregulated, respectively (Fig. S1 and S2). Notably, a core set of 573 genes (521 upregulated and 52 downregulated) showed consistent differential expression when infected valves were compared to both the catheter-injured and control groups (Fig. 3C; Data set S1). These results demonstrate that *S. aureus* infection significantly alters the transcriptional landscape of cardiac valve cells.

**Fig 4:**
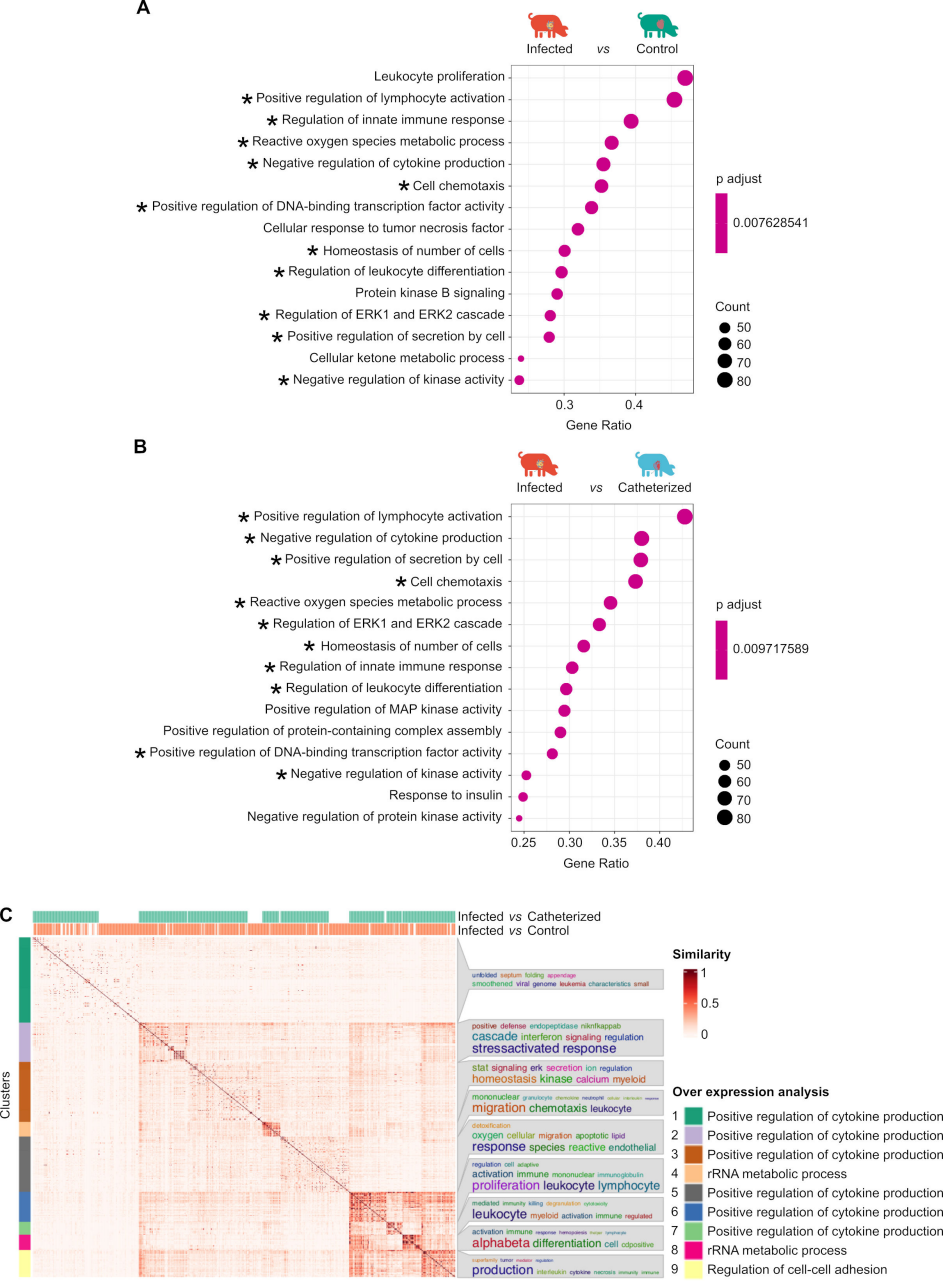
Analysis of differential functional pathways in host valves using the experimental endocarditis porcine model. (**A**) Functional enrichment of differentially expressed genes comparing the infected group to the control group and (**B**) comparing the infected group to the catheterized group. The graphs display the 15 pathways with the most significant differences, ranked by a combined assessment of adjusted *P*-values (FDR) and normalized enrichment scores (NES), following standard GSEA interpretation practices. Of these 15 pathways, those present in both the comparison of infected with control animals and those catheterized are denoted with an asterisk. (**C**) The heatmap includes all gene sets identified as statistically significant. Each row and column represents a gene set, which is ordered based on hierarchical clustering using the “overlapping genes-based relative risk” measure as the distance metric. The figure was generated using the GeneSetCluster web application. The upper portion of the heatmap indicates in which comparison (infected versus catheterized and infected versus control) each gene set is statistically significant. The intensity of the red coloration reflects the degree of similarity between gene sets. Gene sets were grouped into clusters (labeled 1–9, as indicated in the legend), and each cluster was named based on the genes shared across its gene sets. Additionally, a word cloud is displayed, highlighting the most frequently used terms in the descriptions of the pathways associated with each cluster.

DEA and gene set enrichment analysis (GSEA) were performed to identify the functional pathways globally affected in the host during endocarditis in the experimental porcine model. These analyses were performed on the three comparisons made: infected group versus control group, infected group versus catheterized group, and catheterized group versus control group, using the Gene Ontology database (GO) (Fig. 4; Data set S2).

In the valve tissue infected by *S. aureus*, the most significantly affected pathways (ranked by adjusted *P*-value) included those associated with lymphocyte activation, innate immune responses, reactive oxygen species metabolism, cytokine production, cell chemotaxis, and leukocyte differentiation. Additional pathways involved the ERK1/ERK2 cascade, TNF-α signaling via NF-κB, IL-6/JAK/STAT3, IL-2/STAT5, and KRAS signaling, as well as interferon-α/γ responses, allograft rejection, hypoxia, apoptosis, and the p53 pathway (Fig. 4; Data set S2). In contrast, no significant pathway alterations were observed when comparing the control group to the catheter-injured group.

**Fig 5:**
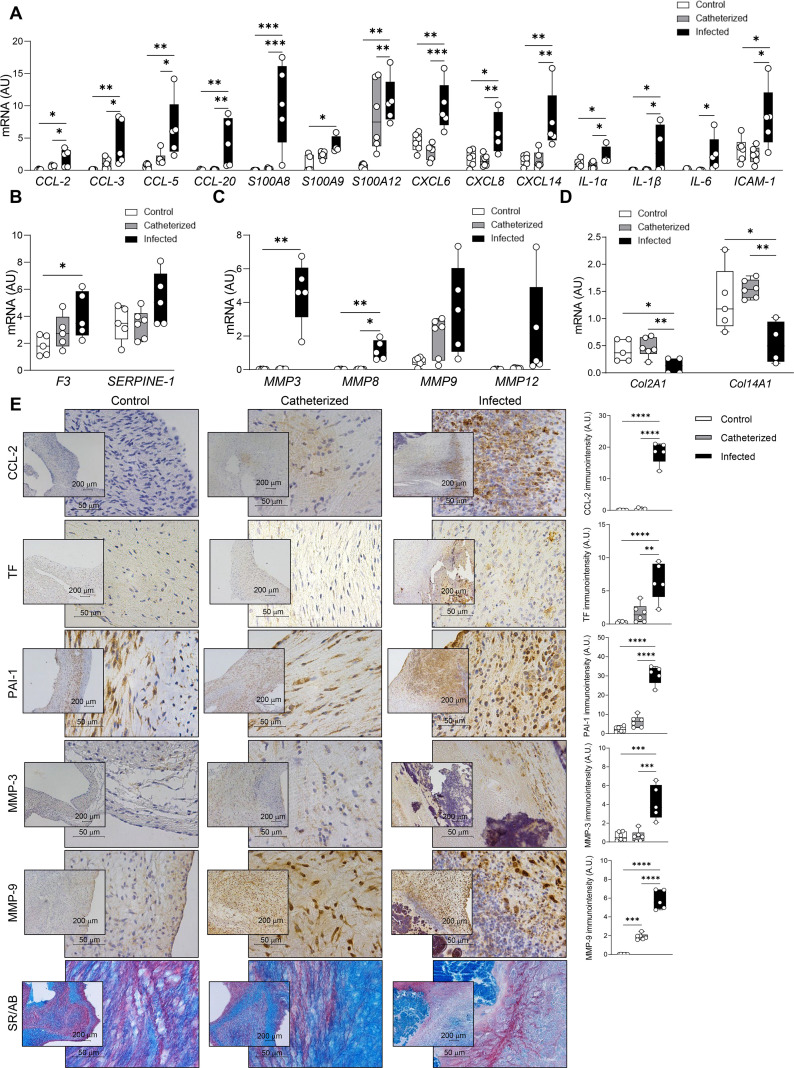
Transcriptional signature of the pig heart valve in *S. aureus*-associated endocarditis. (**A**) mRNA levels of *CCL-2, CCL-3, CCL-5, CCL-20, S100A8, S100A9, S100A12, CXCL-6, CXCL-8, CXCL-14, IL1*α*, IL1*β*, IL-6,* and *ICAM-1*, (**B**) of *F3* and *SERPINE-1*, (**C**) of *MMP-3, MMP-8, MMP-9,* and *MMP-12* and (**D**) of *Col2A1* and *Col14A1* in cardiac valves from the control (*n* = 6, *CCL-2 n* = 4, *CCL-3, S100A9, IL1*α*, IL-6, F3, SERPINE-1, Col2A1* and *Col14A1; n* = 5), catheterized (*n* = 6, *CCL-3, IL-6* and *F3 n* = 5, *S100A9 n* = 4), and infected (*n* = 5, *S100A9*, *CXCL-14*, *IL1*α*, IL1*β*, Col2A1* and *Col14A1; n* = 4) experimental groups. (**E**) Representative images and quantifications of valve sections from control, catheterized, and infected pigs immunostained for CCL-2, tissue factor, PAI-1, MMP-3, MMP-9, and stained with Sirius red/alcian blue. For qPCR analysis data, mRNA levels (expressed in arbitrary units [A.U.]) were normalized to *18S, HPRT, ACTB*, and *GAPDH* levels. Boxplots represent the 25th and 75th percentiles with the median indicated by the center line. Whiskers denote maximum and minimum values. *P < 0.05, **P < 0.01, ***P < 0.001, ****P < 0.0001.

To confirm the transcriptional changes identified by RNA-seq and subsequent functional analyses, key markers were quantified in pig heart valves. It should be mentioned that due to the cutoff points used (log_2_ fold change > 2 or < –2 with an adjusted *P*-value <0.05), the genes *PECAM1*, *CDH5*, *VWF*, *VIM,* and *ACTA2*, which were shown to be differentially expressed in infected animals by qRT-PCR (Fig. 2), do not appear in the transcriptomic results (Data set S1). The expression of several proinflammatory genes *CCL-2, CCL-3, CCL-5, CCL-20, S100A8, S100A9, S100A12, CXCL-6, CXCL-8, CXCL-14, IL-1*α*, IL-1*β*, IL-6,* and *ICAM-1* was significantly increased after *S. aureus* infection (Fig. 5A). In addition, *F3* mRNA was elevated, indicating activation of the coagulation cascade, while *SERPINE1* showed a non-significant upward trend (*P* = 0.08) (Fig. 5B). Dysregulation of matrix metalloproteinases (MMPs) was also observed, with significantly higher levels of *MMP-3* and *MMP-8* in infected valves (Fig. 5C). This increased MMP activity correlated with decreased *Col2A1* and *Col14A1* expression, reflecting compromised collagen integrity (Fig. 5D). Immunohistological analyses confirmed these findings, demonstrating the presence of CCL-2-positive cells surrounding vegetations and increased immunostaining for tissue factor (TF), PAI-1, MMP-3, and MMP-9 in infected valves (Fig. 5E). Double staining with Sirius red/Alcian blue further confirmed the loss of collagen networks in infected animals (Fig. 5E). Taken together, these results suggest that *S. aureus* infection triggers robust inflammatory and coagulation responses, alongside excessive extracellular matrix remodeling and collagen degradation, which ultimately contribute to valve damage.

### Transcriptional signature of the human heart valve in *S. aureus*-associated endocarditis

Once the protocol for simultaneous sequencing of valve and *S. aureus* RNA was successfully established, it was employed on valve samples collected from patients undergoing valve replacement due to acute *S. aureus* endocarditis. Total RNA was extracted from 14 such samples; however, only seven provided sufficient RNA quantity and quality to construct sequencing libraries (Table S4). Of these samples, six were derived from mitral valves, and one (sample H4) was from an aortic valve. Each sample was from a different patient, with the exception of samples H1 and H5, which were mitral valve fragments from the same patient. Capillary RNA electrophoresis showed the presence of *S. aureus* ribosomal RNA, at least in sample H5 (16S/23S), confirming the presence of bacterial RNA and ensuring the detection of *S. aureus* sequences during sequencing (Table S4). After ribosomal RNA depletion, library preparation, and Illumina sequencing, reads were aligned to both the reference human genome (GCA_000001405) and the genome of the *S. aureus* S54F9 strain used in the infection assays. *S. aureus* derived sequences were found only in samples H3 and H5. Although sample H1 came from the same patient as sample H5, it did not yield *S. aureus* sequences, likely reflecting uneven bacterial presence within the valve tissue.

**Fig 6:**
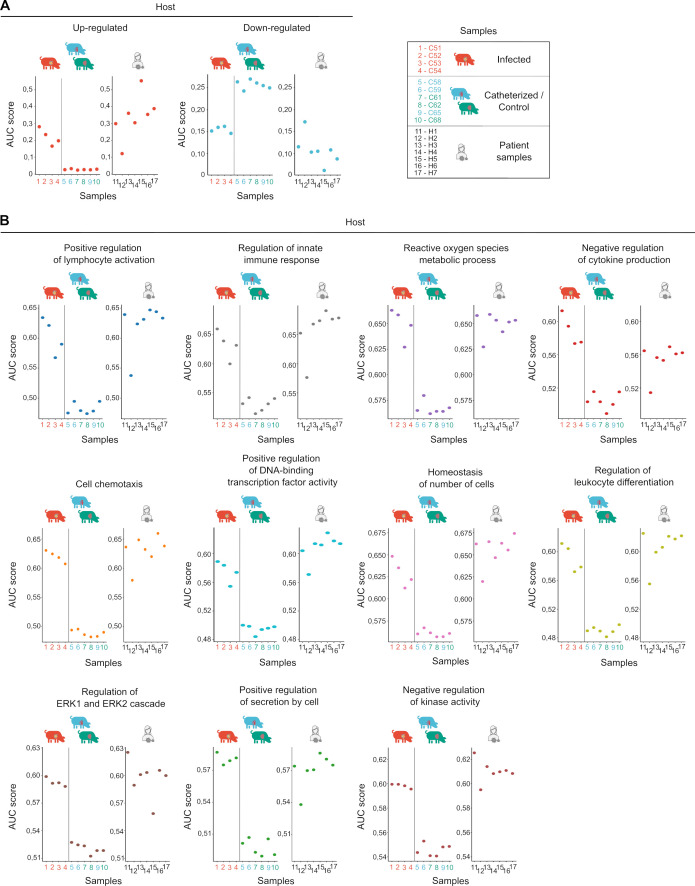
Activity scores of gene sets in pig and patient samples. Gene sets were defined based on differentially expressed genes identified by comparing infected *versus* catheterized pigs in the porcine endocarditis model. We applied an AUC-based activity score approach to evaluate the expression patterns of predefined gene sets in individual RNA-seq samples. The activity score is derived by calculating the AUC from ranked gene expression values within each sample, providing a unitless metric that reflects the relative transcriptional activity of each gene set. (**A**) For each sample, we calculated AUC-based activity scores for both upregulated and downregulated gene sets, using reads mapped to the host genome. Upregulated gene sets are shown as red dots and downregulated gene sets as blue dots. It is important to note that these scores are not intended for direct comparison across different gene sets or species, due to differences in gene set size, expression distribution, and dynamic range. For visualization purposes, some panels display samples from both species side by side; however, valid comparisons should be made only within the same gene set and species under different conditions. (**B**) Activity scores of functional pathways differentially expressed during *in vivo* endocarditis in pigs were calculated for each pig and patient sample. The functional pathways defined in Fig. 4 and Data set S2 were used to compute AUC scores for each sample, as indicated.

**Fig 7:**
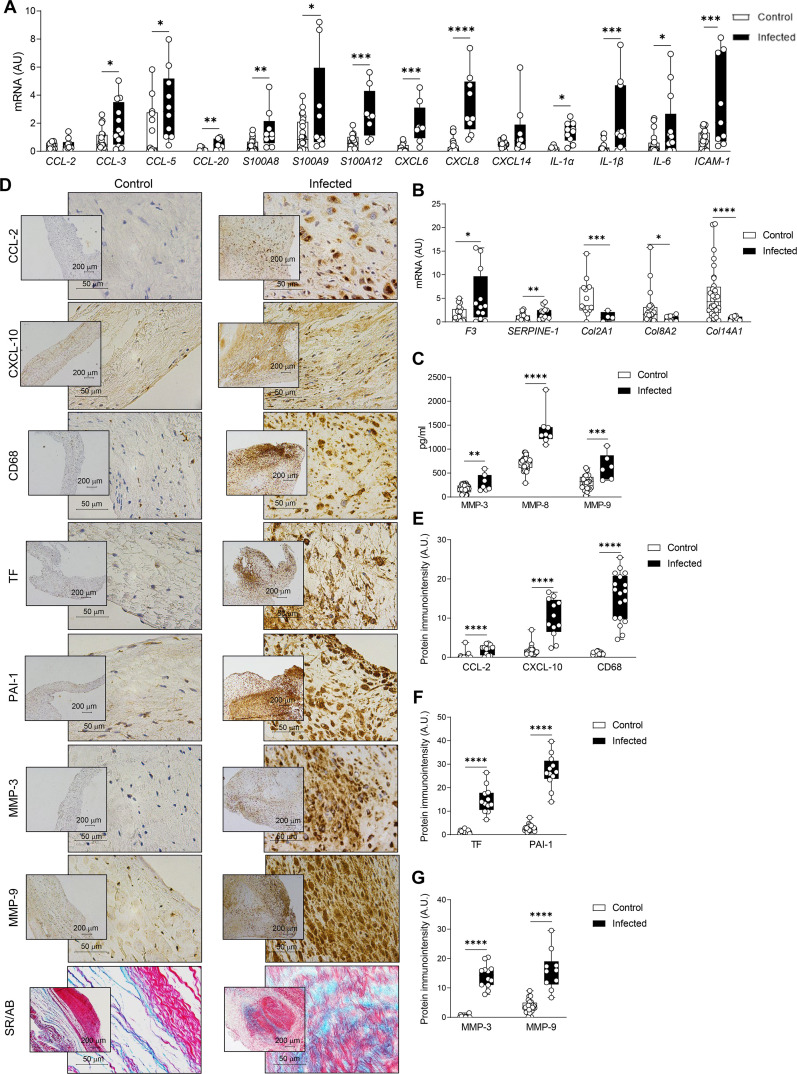
Transcriptional signature of the human heart valve in *S. aureus*-associated endocarditis. (**A**) mRNA expression levels of inflammatory markers: *CCL-2, CCL-3, CCL-5, CCL-20, S100A8, S100A9, S100A12, CXCL-6, CXCL-8, CXCL-14, IL1*α*, IL1*β*, IL-6,* and *ICAM-1*, (**B**) mRNA expression levels of coagulation-related genes: *F3* and *SERPINE1* in cardiac valves from control (*CCL-2 n* = 19*, CCL-3 n* = 20*, CCL-5 n* = 13*, CCL-20 n* = 8*, S100A8 n* = 23*, S100A9 n* = 26*, S100A12 n* = 17*, CXCL-6 n* = 11*, CXCL-8 n* = 26*, CXCL-14 n* = 15*, IL1*α *n* = 9*, IL1*β *n* = 18*, IL-6 and ICAM-1 n* = 23, *F3 n* = 29, *SERPINE1 n* = 25) and IE patients (*CCL-2, CCL-5* and *IL1*α *n* = 10*, CCL-3 n* = 12*, CCL-20*, *S100A12* and *CXCL-6 n* = 8*, S100A8, S100A9, CXCL-8*, *CXCL-14* and *ICAM-1 n* = 9*, IL1*β and *IL-6 n* = 11, *F3* and *SERPINE1 n* = 12). mRNA expression levels of matrix-related genes: *Col2A1, Col8A2,* and *Col14A1* in cardiac valves from control (*ColA1 n* = 14*, Col8A2 n* = 24 and *Col14A1 n* = 32) and IE patients (*Col2A1 n* = 4*, Col8A2 n* = 9 and *Col14A1 n* = 10). (**C**) Protein levels of MMP-3, MMP-8, and MMP-9 quantified using ELISA kits (R&D Systems) in cardiac valves from control (MMP-3 *n* = 30, MMP-8 *n* = 34, MMP-9 *n* = 27) and IE patients (MMP-3 *n* = 7, MMP-8 *n* = 8, MMP-9 *n* = 6). (**D–G**) Representative microphotographs and quantification of immunostaining in valve sections from control and IE patients for CCL-2, CXCL-10, CD68, tissue factor, PAI-1, MMP-3, and MMP-9, along with Sirius red/alcian blue staining. For qPCR analysis data, mRNA levels (expressed in arbitrary units [A.U.]) were normalized to *18S, HPRT, ACTB,* and *GAPDH* levels. Boxplots represent the 25th and 75th percentiles with the median indicated by the center line. Whiskers denote maximum and minimum values. *P < 0.05, **P < 0.01, ***P < 0.001, ****P < 0.0001.

Although we had 34 control human valves from autopsies available (36), which had been previously used for histological, protein, and qRT-PCR analyses (Fig. 2B, E and F), the RNA quality of these samples was not suitable for dual RNA sequencing, likely due to variability in postmortem processing times. For this reason, RNA from these 34 control valves was used solely to validate the sequencing findings. The AUC parameter was used to identify the expression ranking of gene sets that we had previously identified as differentially expressed in the porcine model of IE, this time in the human IE samples. The AUC works by ranking all the genes in a given expression profile and calculating the Area Under the Curve (AUC) of the recovery curve for a specific gene set within that list. The resulting AUC score reflects the coordinated enrichment of that gene set among the most highly expressed genes, independent of absolute expression levels. We refer to this AUC-derived value as the “activity score,” as it indicates the relative transcriptional activity of the gene set in each sample and allows for functional comparisons across conditions. The activity scores in human cells closely mirrored those observed in infected porcine samples, aligning with the clusters of expressed and repressed genes (Fig. 6A). Furthermore, the pathways most affected by *S. aureus* infection in the host, as identified in the pig model (Fig. 4), showed a similar trend in valves from patients with *S. aureus* IE (Fig. 6B). These results suggested that the porcine experimental model mimics cardiac valve alterations found in human IE. To confirm this, additional measurements were conducted on selected markers in human cardiac valves from patients with *S. aureus* IE. The expression of the proinflammatory markers *CCL-3, CCL-5, CCL-20, S100A8, S100A9, S100A12, CXCL-6, CXCL-8, IL-1*α*, IL-1*β*, IL-6,* and *ICAM-1* was significantly elevated in valve samples from patients with *S. aureus* endocarditis as compared to control valves, whereas *CCL-2* and *CXCL-14* were slightly (but not significantly) enhanced (Fig. 7A). Regarding the coagulation profile, both *F3* and *SERPINE1* mRNA levels were enhanced in *S. aureus*-infected valves (Fig. 7B). MMP-3, -8, and -9 expressions were increased in patients with *S. aureus* endocarditis, this increase being accompanied by *Col2A1*, *Col8A2,* and *Col14A1* decreases (Fig. 7B and C). These results were confirmed by the quantification of selected markers in histological samples. Thus, the pro-inflammatory profile found in valves from *S. aureus* IE patients was evidenced by greater CCL-2 and CXCL-10 immunostaining as well as by CD68-positive cells accumulation (Fig. 7D and E). Overactivation of the coagulation cascade was confirmed by increased tissue factor and PAI-1 immunostaining in valves from patients with *S. aureus* IE (Fig. 7DF). Moreover, extracellular matrix remodeling and tissue disarray observed in *S. aureus* infected valves were characterized by greater MMP-3 and -9 immunostaining as well as by collagen disruption found by Sirius red/alcian blue staining (Fig. 7DG).

Taken together, these results demonstrate a strong concordance between the transcriptional profiles of inflammation, coagulation, and MMPs in porcine and human valves, validating the experimental model of *S. aureus*-induced porcine endocarditis at the molecular level.

### Transcriptional signature of *S. aureus* growth on cardiac valves

**Fig 8:**
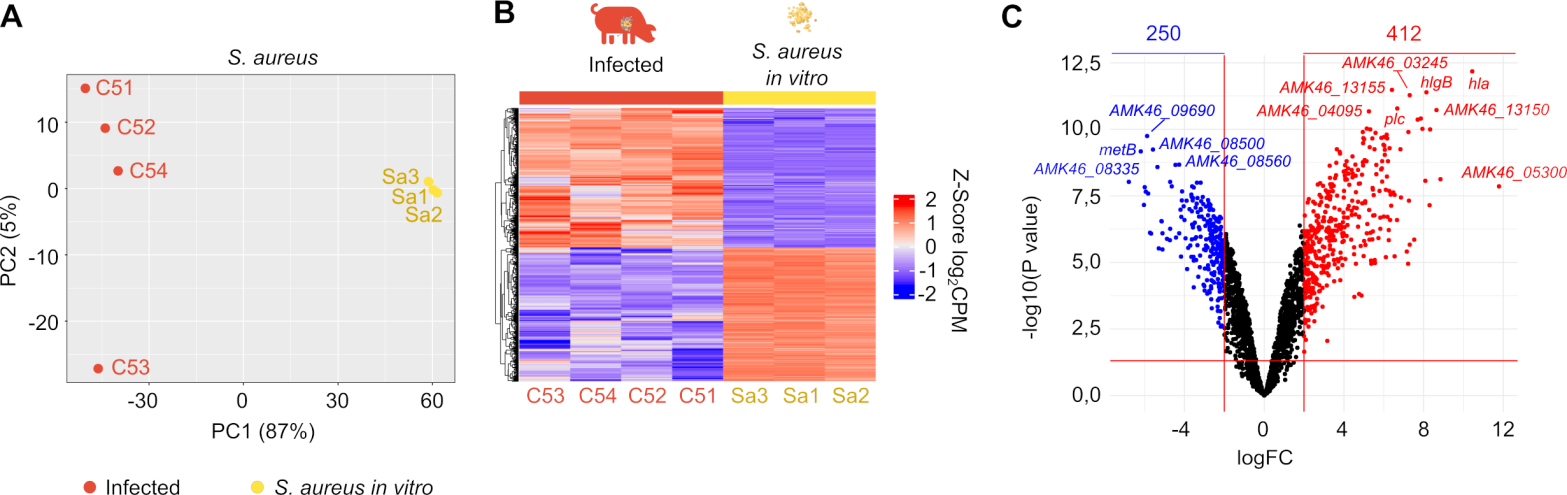
Analysis of differentially expressed genes in *S. aureus* during *in vivo* infection. (**A**) PCA plots of *S. aureus* measurements. The samples are grouped into two categories based on their expression profiles, corresponding to each study group: the infected group and the *S. aureus* S54F9 cultured *in vitro*. The animal numbers for the respective samples are indicated. (**B**) Analysis of differentially expressed genes in *S. aureus* during *in vivo* infection *vs S. aureus* S54F9 *in vitro* cultured samples. The heat maps illustrate the expression profile of differentially expressed genes in each of the specified experimental groups. Genes with a *P*-value adjustment <0.05 are depicted. Colors represent z-scores of voom-transformed log₂-counts-per-million (logCPM) values. Raw counts were normalized using TMM and transformed with voom; each gene’s expression was then centered and scaled across samples, so the color key (–2 to + 2) indicates standard deviations from the mean expression of that gene. The number of animals in each sample and the number of *in vitro* cultures of *S. aureus* (Sa) is also indicated. (**C**) Volcano visualization of differentially expressed genes with a *P*-value adjustment <0.05 and a log_2_ FC >2/<−2. The number of upregulated and downregulated genes is framed and indicated.

**Fig 9:**
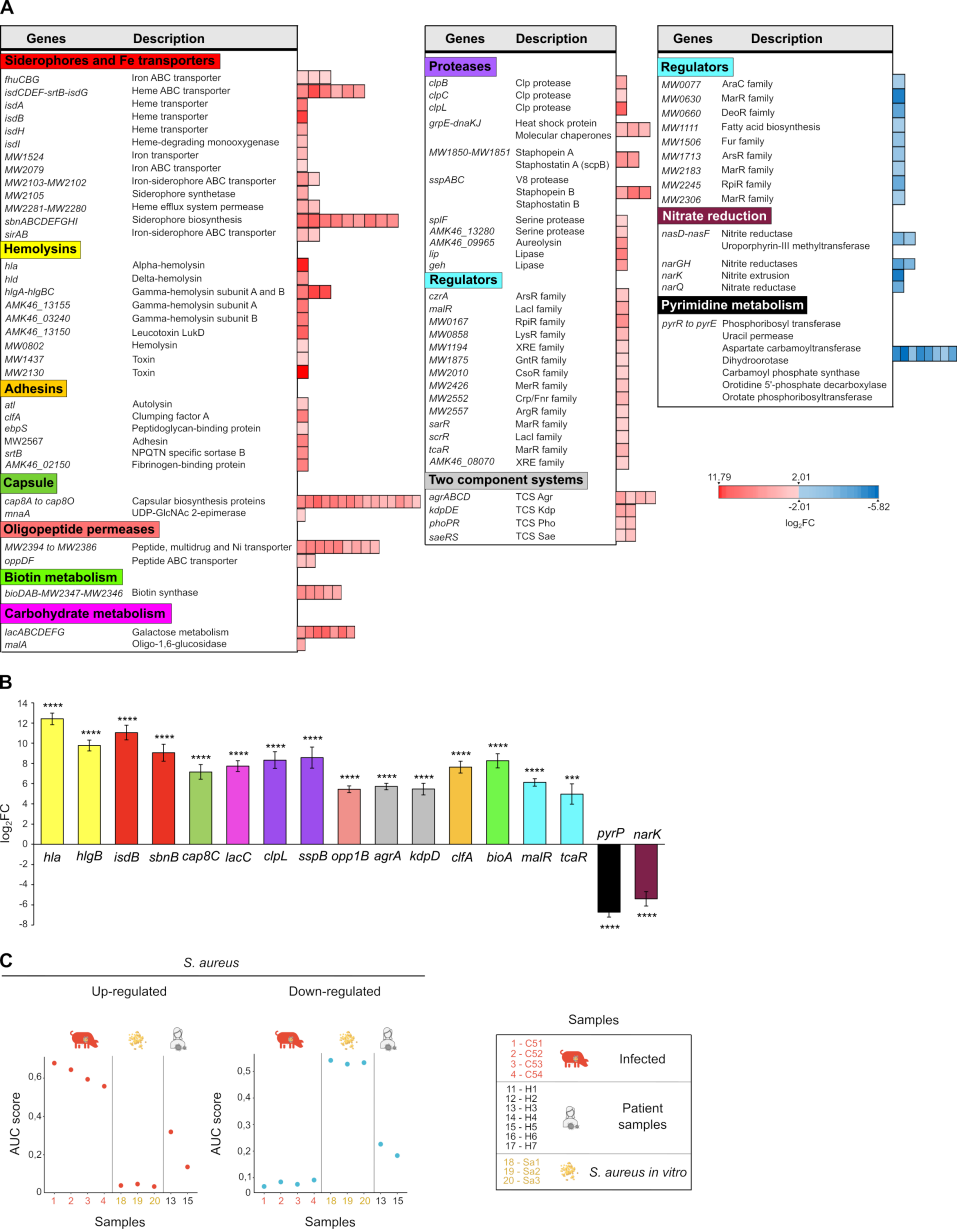
Differential functional pathways expressed by *S. aureus* during endocarditis. (**A**) Heatmap representing upregulated and downregulated functional pathways and genes in *S. aureus* during endocarditis. The log₂ fold change (log₂FC) for each gene is shown, highlighting differential expression between *in vivo* and *in vitro* conditions. (**B**) mRNA expression levels of selected *S. aureus* genes analyzed by qRT-PCR. Data were obtained from infected pig valves (*n* = 4) and *in vitro S. aureus* cultures (*n* = 3). Expression levels were normalized to the housekeeping gene *gyrB* to calculate ΔCt values. ΔΔCt was determined as the difference in ΔCt between *in vivo* and *in vitro* samples. Fold change was calculated using the 2^–ΔΔCt^ method, and the mean log₂FC is presented. Error bars indicate ± standard deviation (SD) of log₂FC values. Statistical significance: ***P < 0.001, ****P < 0.0001. Bar colors indicate functional gene categories: hemolysins (yellow), siderophores and iron transporters (red), capsule biosynthesis (dark green), carbohydrate metabolism (pink), proteases (purple), oligopeptide permeases (light pink), two-component systems (gray), adhesins (orange), biotin metabolism (light green), transcriptional regulators (light blue), pyrimidine metabolism (black), and nitrate reduction (garnet). (**C**) Activity scores of gene sets in pig, patient, and *S. aureus in vitro* samples. Gene sets were defined based on differentially expressed genes identified by comparing *S. aureus* reads from infected pigs in the porcine endocarditis model *versus S. aureus* cultured *in vitro*. Due to the limited number of human samples with sufficient *S. aureus* RNA, we applied an AUC-based activity score approach to evaluate the expression patterns of predefined gene sets in individual RNA-seq samples. The activity score is derived by calculating the AUC from ranked gene expression values within each sample, providing a unitless metric that reflects the relative transcriptional activity of each gene set. For each sample, we calculated AUC-based activity scores for both upregulated and downregulated gene sets, using reads mapped to *S. aureus*. Upregulated gene sets are shown as red dots and downregulated gene sets as blue dots.

To investigate the transcriptional footprint of *S. aureus* during its colonization of the heart valve, we compared the transcriptional profile obtained by sequencing total RNA from infected pig valves (Table S2) with that of the bacteria grown *in vitro* under standard laboratory conditions (TSB medium, 37°C). Reads were aligned to the reference genome of *S. aureus* S54F9 strain, annotated using NCBI’s Prokaryotic Genome Automatic Annotation (PGAAP) (37). The annotated features were genes, CDS, rRNA, tRNA, ncRNA, and repeat regions. UTRs are not annotated. PCA revealed distinct expression patterns between *in vivo* and *in vitro* conditions (Fig. 8A). A subsequent heat map underscored these differences, with *S. aureus* growing within infected valves displaying a markedly different transcriptomic profile compared to bacteria grown in broth culture (Fig. 8B). A volcano plot of the DEA identified 662 genes with altered expression *in vivo*: 412 being upregulated (log_2_ fold change > 2, adjusted *P* value < 0.05) and 250 were downregulated (log_2_ fold change < −2, adjusted *P* value < 0.05) (Fig. 8C). Notably, 152 of these differentially expressed genes encoded hypothetical proteins (Data set S3). Pathway enrichment analysis demonstrated that many of the genes induced in the valve environment were associated with iron uptake and transport (*sbnABCDEFGHI*, *sirAB,* and *isdABCDEF-srtB-G*), adhesins (*clfA, ebpS, and atl*), hemolysins (*hlgABC, hld, lukD,* and *alpha-hemolysin*), proteases (*clpBCL, splF,* and *sspABC*) and lipases (*geh*), capsule synthesis (*cap8ABCDEFGHIJKLMNO*), biotin metabolism (*bioDAB*), and various regulatory circuits including two-component systems (*agrABCD, phoPR, saeRS,* and *kdpDE*) and other regulators (*sarR, tcaR, czrA, malR,* and *scrR*) (Fig. 9A; Data set S3). Conversely, genes involved in pyrimidine metabolism (*pyrRPBC-AA-AB-FE*) and nitrate respiration (*narQ, narK, nasD, nasF, narG,* and *narH*) were downregulated in the valve environment. These results were validated by measuring the mRNA levels of selected genes using qRT-PCR (Fig. 9B).

In human valve samples, the abundance of *S. aureus* RNA was highly variable, likely reflecting the effects of intensive antibiotic treatment on bacterial load, as well as non-uniform pathogen distribution within the tissue. In the human samples with sufficient *S. aureus* RNA (H3 and H5), the bacterial transcriptome closely resembled its profile observed in porcine heart valves (Fig. 9C; Fig. S3), with the exception of one sample (H5), which showed differences in gene sets related to capsule synthesis, toxins, and two-component systems (Fig. S3). Overall, these findings indicate that *S. aureus* adopts a highly selective gene expression strategy during growth on the heart valve, regulated by multiple factors and two-component systems, to optimize iron acquisition, tissue adhesion, protein turnover, immune evasion, carbohydrate metabolism, and external polysaccharide composition. This integrated response facilitates successful colonization of native heart valves and contributes to the pathogenesis of IE.

## DISCUSSION

Investigating the dynamics of endocarditis with human samples presents significant challenges as access to patients’ cardiac valves is limited to cases requiring valve replacement surgery. This procedure is usually reserved for situations where patients have exhausted other therapeutic options and have been treated with high doses of antibiotics prior to replacement. These limitations highlight the need to develop experimental infection models that closely mimic clinical scenarios. In this study, we use a porcine model chosen for its close anatomical, physiological, and immunological similarity to humans (34, 38). Within the spectrum of IE disease development, the animal model we present is characterized by being in an acute phase of the infection, with symptoms compatible with IE at the time of sampling. Several aspects of our infection model are worth highlighting (i) the valves of all infected pigs had a bacterial load sufficient to collect bacterial RNA for analysis; (ii) catheter injury, which facilitates *S. aureus* colonization, induced minimal changes in the transcriptional profile of the cells when compared to those in the control group; (iii) histological and molecular changes induced by *S. aureus* closely mirrored those observed in infected human valve samples; and (iv) the transcriptomic profile of *S. aureus* in pig heart valves was consistent with data obtained in previous studies in rat valve vegetations, mouse kidney, and human abscesses (27, 39).

Recent studies investigating the early stages of *S. aureus* adhesion to valve surfaces have shown that the adhesion mechanism depends on the nature of the predisposing factor, whether it is valvular damage or an inflammation (18). In cases of mechanical valve damage, *S. aureus* binds directly to von Willebrand factor (vWF) and fibrin deposited on the damaged tissue through its surface adhesins, with platelets not playing a role in this process. Conversely, when endothelial inflammation occurs, vWF is released, which attracts platelets. These platelets, in turn, recruit bacteria to the endothelial surface, thereby facilitating bacterial adhesion. Our experimental model of bacteremia following catheter-induced endothelial damage of heart valves supports and extends these findings. Endothelial disruption exposes collagen and other matrix molecules that promote fibrin deposition and platelet accumulation. While transcriptomic data indicated no inflammation in mechanically damaged valves alone, infection triggered substantial inflammation, dysregulated coagulation, and increased matrix metalloproteinase (MMP) activity. This led to vegetation formation, structural damage to the valves, and significant tissue disarray. Moreover, the presence of activated macrophages in damaged tissues elevated proinflammatory cytokines, enhanced tissue factor and fibrin deposition, and increased MMP activity, collectively exacerbating valvular destruction and predisposing to embolization (40, 41). These observations align with previous reports indicating that serum cytokine levels can reflect the severity of IE (42). Our results also suggest that host-derived proteases contribute significantly to tissue damage induced by *S. aureus*, consistent with prior evidence that proteases present in vegetations predominantly originate from the host (43). In line with our findings in both experimental and human IE, a previous study analyzing cardiac valves from five patients with IE caused by *S. aureus* or *Streptococcus* species identified a large number of upregulated genes involved in chemotaxis, including CXC and CC chemokines, as well as MMPs (44).

The majority of the *S. aureus*-induced histological and molecular alterations closely resembled those observed in infected human valve samples. However, vimentin expression followed a different pattern. Vimentin is typically located in the cytoplasm but can also be secreted as an extracellular protein. Several bacterial factors are known to interact with vimentin, regulating its assembly and disassembly through post-translational modifications (45). To our knowledge, this is the first report of vimentin alterations in the context of *S. aureus*-induced IE. In the pig model, vimentin levels decreased, whereas in human IE valves, they increased. A plausible explanation is that, in the pig model, the decrease reflects rapid filament disassembly prior to their release into the extracellular space during the acute phase of infection. In contrast, the elevated vimentin levels observed in human IE likely reflect the chronic inflammatory state, characterized by continuous vimentin synthesis driven by pro-inflammatory cytokines.

With regard to the pathogen’s perspective, our findings indicate that the *S. aureus* transcriptome on the heart valve surface is characterized by heightened heme iron acquisition, evidenced by the overexpression of IsdABCDEFGH-SrtB proteins. Additionally, iron uptake from siderophores such as lactoferrin and transferrin is facilitated via staphyloferrin B (46). IsdB, a surface-exposed receptor, plays a pivotal role in extracting iron from human hemoglobin and also interacts with integrins and key extracellular matrix components, including von Willebrand factor and vitronectin (47–49). Consistent with previous studies, our results show that ClfA induction is critical for endocarditis. However, we did not observe induction of fibronectin-binding proteins (FnBPA and FnBPB) that have been noted to play a significant role in platelet activation, contributing to vegetation and thrombus formation on the valve surface (50). Since ClfA can also activate platelets, it may compensate for the absence of FnBPAB induction. Other proteins previously associated with endocarditis in murine models, such as von Willebrand factor binding protein (vWbp) and sortase A (SrtA), have demonstrated reduced colonization capacity when mutated in a rat model of damage-induced endocarditis (18, 51). However, in our porcine model, we did not observe significant changes in the expression of these proteins. One possible explanation is that baseline SrtA levels are already sufficient to anchor surface proteins, thus obviating the need for induced expression. We cannot exclude the possibility that the lack of FnBPAB, vWbp, and SrtA induction is specific to the *S. aureus* S54F9 strain; however, this strain is well-established as a model for studying endocarditis due to its strong propensity to cause the disease.

Another key observation from our analyses is that *S. aureus* induces the expression of the capsule operon. Furthermore, cobalt and nickel transporter permeases (Opp1ABCDF) are consistently upregulated *in vivo*, suggesting that the cardiac valve environment imposes limitations on these essential metal ions, triggering their selective induction (52, 53).

Several proteases, hemolysins, and toxins were also upregulated *in vivo*, notably alpha (Hla), delta (Hld), gamma (HlgABC) hemolysins, and the leukocidin LukD. These hemolysins play a critical role in endocarditis pathogenesis by facilitating the initial colonization of heart valves, extracting heme as a nutrient source through red blood cell lysis, promoting tissue invasion, and aiding in immune evasion. Additionally, other proteins potentially functioning as toxins and hemolysins, though not yet characterized, were induced during infection (Fig. 9; Data set S3).

Among the most prominently altered transcripts were those encoding regulatory proteins, including numerous hypothetical proteins bearing domains characteristic of regulatory factors. The large number of hypothetical regulatory proteins showing specific expression changes in the valve environment underscores the importance of studying bacterial gene regulation under *in vivo* conditions. In these complex settings, genes that remain inactive under laboratory conditions may become pivotal contributors to pathogenic processes. Another key group of genes essential for adapting to the valve environment is those encoding proteins of two-component systems (TCS). Among the 16 TCS present in *S. aureus*, we detected overexpression of only four: Agr and Sae, which are known to govern a wide range of virulence factors, and Kdp and Pho, which play roles in potassium uptake and responses to low-phosphate conditions, respectively.

Comparison of our results with previously published *S. aureus* transcriptomes from other infection models reveals that certain biological processes are commonly induced across diverse infection sites (54, 55). These include genes involved in iron uptake, through both heme acquisition and siderophore systems, expression of toxins and hemolysins that drive tissue invasion and immune evasion, and activation of key two-component regulatory systems such as Agr and Sae. In contrast, endocarditis exhibits several unique transcriptional signatures. These include strong induction of the capsule operon, which tends to remain stable or even be downregulated in other tissues; upregulation of cobalt/nickel transporter permeases (Opp1ABCDF), suggesting a metal-limited microenvironment in the heart valves; and marked overexpression of ClfA, which appears sufficient to mediate adhesion in the absence of FnBPA/B. Additionally, while other infection sites often activate the SrrAB system in response to hypoxia, our data show overexpression of Kdp and Pho, indicating that the valve environment is nutrient-limited but not severely hypoxic. Future studies should evaluate the infectivity of mutants in genes that are strongly induced during the endocarditis process to identify potential targets whose inhibition could facilitate treatment of the infection.

Taken together, our results provide a detailed representation of the transcriptional profile of *S. aureus* during endocarditis in a porcine infection model and suggest a strong parallel with the transcriptional changes occurring in human heart valves during acute endocarditis. Importantly, this study highlights the value of integrating data from both the porcine model and human samples. While our analysis cannot exclude the possibility of additional mechanisms unique to human infections that may not be captured in the pig model, the key pathophysiological processes identified in the animal model appear to be conserved in human endocarditis.

### Limitations

Our study has several limitations that warrant consideration. First, we evaluated only a single time point in the porcine model of endocarditis. Due to the severity of the infection, extending the analysis to later stages is challenging, limiting our ability to determine how bacterial and host gene expression profiles evolve over time as the immune response intensifies. Additionally, our human patient cohort exhibited heterogeneity in terms of age, sex, and race, reflecting the real-world clinical diversity of IE. The fact that patients had undergone intensive antibiotic treatment before valve replacement may have influenced both bacterial and host transcriptional signatures, potentially obscuring certain aspects of the infection process. Nevertheless, such diversity is clinically relevant. Finally, the pathological diagnosis is inherently retrospective, as it was made only in patients who ultimately required valve replacement.

## MATERIALS AND METHODS

### Animals and housing

Eighteen clinically healthy female Landrace-Large White crossbred pigs (body weight 19–27 kg, equivalent to 8–10 weeks of age), obtained from our breeding center, were used in this study and were housed in the animal facilities of the University of Navarre in accordance with all governmental and institutional laws, guidelines, and regulations. The pigs were acclimatized for 7–10 days before the start of the study. They were fed with a pressed pellet diet (antibiotic-free) from a commercial supplier to maintain a steady and appropriate growth curve and given water *ad libitum*, except for 6–8 hours of fasting prior to procedures requiring anesthesia.

### Animal model of endocarditis in pigs

The porcine model of *S. aureus* IE was adapted from a previous study by Christiansen et al. (33) with specific modifications. Following a 10-day acclimatization period, 18 pigs aged 8–10 weeks (19–27 kg) were divided into three experimental groups: control (*n* = 6), catheterized (*n* = 6), and infected (*n* = 6). Six animals received no treatment (control group), while 12 animals had a radiopaque Flex percutaneous introducer sheath (Arrow CL-07824 Super Arrow-Flex percutaneous sheath introducer set, 8F, 24 cm) inserted via dissection into the left carotid artery, extending to the left ventricle (catheterized and infected groups, day 1), remaining in place until sacrifice. Forty-eight hours later (day 3), five of these pigs (note that one pig of the infected group died at day 2) were intravenously inoculated with porcine *S. aureus* strain S54F9 (37) at a dose of 10^7^ cfu/kg (infected group), while six were sham inoculated with sterile isotonic saline (catheterized group). Daily observation recorded clinical signs post-infection. Twenty-four hours post-infection (day 4), infected animals displayed mucus, fever, and papules. By 48 hours post-infection, all infected animals exhibited high fever, pustules, and immobility, reaching humane endpoint criteria, leading to euthanasia (day 5). Electrocardiography monitored the introducer sheath implantation during the procedure, with correct positioning confirmed by electrocardiogram changes during manipulation and transthoracic echocardiography post-procedure. Blood samples for bacterial culture were collected on days 1, 3, and 5. Transesophageal echocardiography was performed on the day of sacrifice (day 5) in the catheterized and infected groups. At sacrifice, cardiac tissue samples (aortic, mitral, pulmonary, tricuspid valves, and left ventricle injured by the introducer sheath) were isolated, with analyses focused on aortic valves (AVs). Samples for molecular studies were snap-frozen and ground into powder, divided equally for transcript and protein analysis, ensuring accuracy and reliability of the quantitative data. All samples were stored at −80°C until processing.

### Surgical procedure, inoculation, and euthanasia

Animals received pre-medication with 4 mg/kg tiletamine/zolazepam (Zoletil 100, VIRBAC ESPAÑA S.A.) administered intramuscularly (IM). A 22-G intravenous (IV) catheter was inserted into the marginal ear vein, and multiparameter connections were established to monitor vital signs. Following sedation, animals were administered 3 mg/kg propofol (Propofol Lipuro 10%, B. BRAUN) IV, underwent endotracheal intubation, and were mechanically ventilated with supplemental oxygen. During surgery, anesthesia was maintained with a continuous infusion of 14 mg/kg/h propofol (Propofol Lipuro 20%, B. BRAUN) and 2% isoflurane (IsoVET, B. BRAUN) inhalation, while analgesia was sustained by continuous intravenous infusion of 0.03–0.06 mg/kg/h remifentanil (Ultiva 2 mg, Aspen) and application of a fentanyl patch (Durogesic Matrix 12 µg/h, JANSSEN-CILAG) behind the ear. Animals were positioned supine, and the ventral neck area was prepared aseptically. The left internal carotid artery (CA) was surgically exposed via a vertical incision lateral to the trachea, with subsequent dissection of the strap muscles. Two 2/0 ligatures (Polysorb 2/0, COVIDIEN) were placed around the CA, with the distal end permanently ligated. An 8F introducer sheath (Arrow CL-07824 Super Arrow-Flex Percutaneous Sheath Introducer Set, 8F, 24 cm) was trimmed to 6 cm with a no. 23 blade and inserted into the CA until crossing the aortic valve, monitored electrocardiographically. The introducer was clamped with a proximal ligature, and the incision was closed with absorbable suture (Vicryl 2/0, Ethicon). The protruding end of the introducer was secured to the pig’s neck. All surgical procedures were performed under aseptic conditions, with pigs monitored until fully awake. Transdermal fentanyl (Durogesic Matrix 12 µg/h, JANSSEN-CILAG) provided postoperative analgesia. Two days post-introducer implantation, pigs were inoculated intravenously with a prepared bacterial inoculum. The *S. aureus* S54F9 strain was cultured, and colonies were resuspended in 1× PBS to achieve an OD at 600 nm of 0.2, corresponding to 10^8^ cfu/mL. Serial dilutions were plated for enumeration, with each pig receiving an appropriate volume until reaching a dose of 10^7^ cfu/kg, followed by saline flushing. Animals in the catheter group underwent the same procedure but received saline only. Daily observations recorded clinical signs post-infection, with fever treated using 4 mg/kg carprofen (NOROCARP 50 mg/mL, Karizoo) subcutaneously. After 48 hours post-infection, animals were euthanized using anesthesia and KCl (potassium chloride 15%, B. BRAUN) intravenously to stop the heart in diastole. Necropsy was conducted, and cardiac tissue samples (aortic, mitral, pulmonary, tricuspid valves, and left ventricular myocardium) were collected. Samples were immediately frozen in liquid nitrogen for subsequent RNA extractions and kept at −80°C until processing.

### Echocardiography

Transthoracic echocardiograms (TTE) were conducted on all animals, with assessments performed by the same experienced sonographer on the day of catheter placement (day 1), inoculation (day 3), and sacrifice (day 5). Pigs were positioned supine on the examination table after anesthesia induction as described earlier. Alcohol and ultrasound gel were applied to ensure optimal probe-skin contact. Right parasternal two-dimensional long-axis and short-axis views were utilized to capture multiple tomographic planes, supplemented by spectral Doppler and color flow imaging. Prior to catheter insertion, all animals exhibited normal left ventricular ejection fraction, lacked structural abnormalities, and demonstrated normal aortic valve function. Post-infection, meticulous examination focused on structures adjacent to the catheter for irregularly shaped echogenic masses adhering to heart valves or the mural endothelial surface. Evaluation for new valve regurgitation was performed using color flow imaging of left-sided heart valves. Echocardiographic data were digitally stored for subsequent analysis employing specialized software. Additionally, esophageal echocardiography (EET) was conducted on animals in the catheter and infected groups at sacrifice (day 5) to identify potential signs of IE.

### Bacteriology: examination of blood

Blood samples for bacterial culture were collected on the day of catheter placement (day 1), inoculation (day 3), and sacrifice (day 5). In the control group, blood collection occurred solely on the day of euthanasia, and in the catheterized group, samples were obtained only on the day of catheter placement (day 1) and euthanasia (day 5). Ten milliliters of blood was drawn from the femoral vein and combined with BD BACTEC Plus Aerobic medium in plastic culture tubes (Becton Dickinson, Ref. 442023) and incubated at 37°C for 5 days. Bacterial growth was assessed using BD BACTEC FX (Becton Dickinson) equipment, and identification of the microorganism(s) cultured in the enrichment medium was conducted using MALDI Biotyper (BRUKER).

### *S. aureus* PCR in heart valves

Cardiac tissue samples (aortic and mitral valves) from all animals were processed to amplify the 16S rRNA of *S. aureus* by PCR. DNA was extracted from the samples using the kit MagCore Cartridge 102 (RBC Bioscience). Subsequently, the PCR of the samples was carried out using the oligos fD1-1 (AGAGTTTGATCCTGGCTCAG)/rD2 (ACGGCTACCTTGTTACGACTT). Positive PCRs were determined by the presence of a band of the size corresponding to the amplicon in a 2% agarose gel. Bands were purified and sequenced (STAB VIDA) using a fD1-1 primer.

### Heart valves of patients with endocarditis due to *S. aureus*

Fourteen patients undergoing surgical aortic or mitral valve replacement at our institution were enrolled between April 2018 and November 2023 for *S. aureus* IE, following current clinical practice guidelines (2). Healthy control valves (*n* = 34) were procured from autopsies (36).

Diagnosis of IE was based on positive blood cultures for *S. aureus*, along with clinical and echocardiographic data assessed using the Duke score, confirming the diagnosis of *S. aureus* IE. Likewise, culture and/or PCR of biopsied valves confirmed *S. aureus* endocarditis diagnosis. Patients received antibiotic treatment from diagnosis until surgical removal. Tissue samples were frozen in liquid nitrogen post-valve surgery and stored at −80°C until processing.

### RNA extraction from heart valve tissue samples for transcriptomic assays

Frozen tissue samples were weighed without thawing and ground with a pestle in liquid nitrogen. The resulting frozen powder was then resuspended in denaturing Solution D (Sigma 30911-100ML) with 0.1 M β-mercaptoethanol at a ratio of 1 mL per 90 mg of tissue. This resuspended tissue powder underwent lysis twice on ice at speed 6 using a hand-held homogenizer (D1000 Benchmark) equipped with a sawtooth generator head 7 × 50 mm (D1000-M7). Total RNA was extracted from valve tissue samples using the TRIzol reagent method (Invitrogen) as described (56). Briefly, 460 µL of the homogenate was transferred to Lysing Matrix E tubes (MP Biomedicals) containing 500 µL acid phenol (Invitrogen) and mixed. The number of tubes used varied based on sample volume. Samples were lysed using the Fastprep apparatus (MP Biomedicals) at speed 6.0 for 45 seconds at 4°C, repeated twice, with 1-min intervals on ice between cycles. The lysate was then centrifuged at 14,000 rpm and 4°C for 10 minutes. The resulting aqueous phase was transferred to 2 mL tubes containing 1 mL TRIzol, mixed, and incubated for 5 minutes at room temperature. Subsequently, 100 µL chloroform was added, gently mixed, and incubated for 3 minutes at room temperature, followed by centrifugation at 14,000 rpm for 10 minutes at 4°C. The resulting aqueous phase was transferred to another 2 mL tube containing 200 µL chloroform, mixed, and incubated for 5 minutes at room temperature, followed by centrifugation at 14,000 rpm for 5 minutes at 4°C. The resulting aqueous phase was transferred to a 2 mL tube containing 500 µL isopropanol, mixed by inversion, and incubated for 15 minutes at room temperature to precipitate the RNA. The tube was then centrifuged at 14,000 rpm for 15 minutes at 4°C. All dried RNA pellets from one sample were combined and resuspended in 168 µL DEPC-treated water.

This RNA solution underwent treatment with 10 µL TURBO DNase (Invitrogen) in the presence of 2 µL SUPERase-In RNase inhibitor (Invitrogen) at 37°C for 30 minutes. Subsequently, 100 µL DEPC-treated water was added to the DNA-free RNA solution, which was then transferred to a tube containing 300 µL acid phenol, mixed, and incubated for 3 minutes at room temperature. The tube was centrifuged at 14,000 rpm for 5 minutes at 4°C. The resulting aqueous phase was transferred to a tube containing 300 µL chloroform, mixed, and centrifuged at 14,000 rpm for 5 minutes at 4°C. The aqueous phase was then transferred to a tube containing 200 µL ammonium acetate 5 M and 1 mL ethanol 96%, mixed by inversion, and incubated at −20°C for 2 hours to precipitate the RNA. The tube was centrifuged at 14,000 rpm for 30 minutes at 4°C, and the RNA pellets were resuspended in 14 µL DEPC-treated water.

RNA concentration was determined by spectrophotometry, and RNA quality was assessed by capillary electrophoresis using the Agilent RNA 6000 Nano Kit and Bioanalyzer instrument (Agilent). RNA samples were stored at −80°C until further use.

Total RNA extraction from *S. aureus* grown *in vitro* followed a similar procedure. *S. aureus* strain S54F9 was cultured overnight in 5 mL TSB at 37°C under shaking conditions (200 rpm). The cultures were then diluted 1:100 in 80 mL TSB and incubated in 500 mL flasks at 37°C under shaking conditions (200 rpm) until reaching an OD_600_ of 0.8. Bacterial cultures were then centrifuged, and the resulting pellets were frozen in liquid nitrogen and stored at −80°C until required. For total RNA extraction, bacterial pellets were resuspended in 400 µL solution A (glucose 10%, Tris 12.5 mM, pH 7.6, and EDTA 10 mM) and mixed with 60 µL 0.5 M EDTA. The resuspended cells were transferred to Lysing Matrix B tubes (MP Biomedicals) containing 500 µL acid phenol (Invitrogen). Bacteria were lysed twice using the Fastprep apparatus at speed 6.0 for 45 seconds at 4°C, with 1-minute intervals on ice between cycles, followed by centrifugation at 14,000 rpm and 4°C for 10 minutes. The resulting aqueous phase was processed further following the same steps as for tissue samples. RNA concentration and quality assessment for bacterial samples followed the same protocol as for tissue samples. RNA samples were stored at −80°C until further use.

### Construction of cDNA libraries and sequencing by Illumina

The cDNA library construction and sequencing by Illumina were performed by the company Vertis Biotechnologie AG and the Core Unit Systems Medicine of the University of Würzburg. The same protocol was followed to prepare the libraries of each of the samples analyzed. Ribosomal RNA molecules (porcine/human/bacteria) were depleted from the total RNA samples using a mixture of siPOOLs human and PANProkaryote rRNA removal solutions (siTOOLs Biotech). The ribodepleted RNA samples were first fragmented by ultrasound (1 or 4 pulses of 30 s each at 4°C). An oligonucleotide adaptor was then ligated to the 3′' end of the RNA molecules. First-strand cDNA synthesis was performed using M-MLV reverse transcriptase and the 3′ adapter as primer. The first-strand cDNA was purified, and the 5′ Illumina TruSeq sequencing adapter was ligated to the 3′ end of the antisense cDNA. The resulting cDNA was PCR amplified to approximately 10–20 ng/μL using a high-fidelity DNA polymerase. The cDNA was purified using the Agencourt AMPure XP kit (Beckman Coulter Genomics) and analyzed by capillary electrophoresis. For Illumina NextSeq sequencing, the samples were pooled in approximately equimolar amounts. The cDNA pool was size-fractionated in the 200–500 bp/200–600 bp size range using a preparative agarose gel. An aliquot of the size fractionated pool was analyzed by capillary electrophoresis. Primers used for PCR amplification were designed for TruSeq sequencing according to the instructions of Illumina. The following adaptor sequences flanked the cDNA inserts: TruSeq_Sense_primer i5 Barcode 5′-AATGATACGGCGACCACCGAGATCTACACNNNNNNNNACACTCTTTCCCTACACGACGCTCTTCCGATCT-3′ TruSeq_Antisense_primer i7 Index 5′-CAAGCAGAAGACGGCATACGAGATNNNNNNNNGTGACTGGAGTTCAGACGTGTGCTCTTCCGATCT-3′. The cDNA pool was sequenced on an Illumina NextSeq 500 system using 75 bp–101 bp read length.

### Dual RNA-Seq data analyses

After a quality analysis of the fastqs of the samples using FastQC (version 0.11.7) and MultiQC (version 1.7) (57), adapters were removed from the reads through Timomatic (version 2.6.0a) (58). Once the correct extraction of adapters and the good quality of the reads were confirmed, the alignment to the reference genomes continued. The alignment of each sample was first performed with the reference genome of the host species: *Homo sapiens* and *Sus scrofa* (GRCh38 and Sscrofa11.1, respectively) and was carried out through STAR (version 2.6.0a) (59) with the default values. Next, those reads that did not map to the reference genome were aligned with Bowtie2 (version 2.2.3) (60) to the *S. aureus* S54F9 bacterial reference genome (ASM128160v1). To annotate these alignments, the following GTFs were used, respectively: GRCh38.83, Sscrofa11.1.104, and ASM128160v1, respectively. *S. aureus* samples grown *in vitro* were exclusively mapped against the *S. aureus* S54F9 bacterial reference genome (ASM128160v1).

### Filtering and normalization

The samples extracted from the valves of *S. scrofa* and *H. sapiens,* and of *S. aureus* grown *in vitro,* were run in parallel throughout the process. First, the low-expressed genes were filtered to obtain those genes with an ensemble ID that had at least one count in at least two samples. Sample normalization was then performed using the edgeR R package (version 3.32.1) (61). In this way, a total of 10,630 *H. sapiens,* 12,845 *S*. *scrofa,* and 2,295 *S*. *aureus* genes were retained in the valve samples.

### DEA and GSEA

DEA was conducted for each group. Specifically, DEA was performed on three experimental groups of *S. scrofa*: infected vs control, infected vs catheterized, and catheterized vs control. Additionally, a DEA was conducted on bacterial readings obtained from infected aortic valves (infected vs *in vitro*). These analyses focused on genes that passed the filtering criteria, utilizing the limma R package (version 3.46.0) (62), employing default settings and an FDR cutoff of 0.05.

Subsequently, for GSEA of the samples, the ensemble ID of *S. scrofa* and *S. aureus* strain S54F9 were converted into the symbol ID of *H. sapiens* and *S. aureus* strain 502A, respectively. Orthologous IDs of the differentially expressed IDs of *S. aureus* strain S54F9 were also identified in *S. aureus* strains MW2 and NCTC8325 (Data set S3). This conversion enabled GSEA using the gseKEGG function of the R package clusterProfiler (version 3.18.1) (63), configured with minGSSize = 3 and maxGSSize = 100. Additionally, for *S. scrofa*, gseGO was employed with the same configuration. GeneSetCluster web application was used to cluster and visualize the gene sets identified in the GSEA derived from the contrast in *S. scrofa* when comparing the infected vs control and infected vs catheterized comparisons (64, 65).

### Gene set activity scoring in bulk profiles

To investigate the score of a given gene set within a bulk sample, we used the area under the curve analysis from the AUCell R package (version 1.12.0) (66). The gene sets investigated in the *S. scrofa* samples were both the upregulated genes (log_2_FC ≥ 1) and the downregulated genes (log_2_FC ≤ −1) derived from the infected vs catheterized contrast. As with the *S. aureus* samples in the comparative infected vs *in vitro* analysis, the AUC was calculated in human valve samples for both *H. sapiens* and *S. aureus*.

### Real-time reverse transcription PCR

To analyze the expression of pig and human genes by qRT-PCR, total RNA was extracted from cardiac valve tissues using guanidinium thiocyanate (PRImeZOL, Canvax). First-strand cDNA was synthesized using the Script Advanced cDNA Synthesis Kit for RT-qPCR (BioRad) according to the manufacturer’s instructions. Quantitative PCR analysis was performed with iQ SYBR Green Supermix (BioRad) on a CFx Connect Real Time System (BioRad). Relative gene expression was quantified using the comparative Ct method, normalizing the expression of target genes to the Ct values of the housekeeping genes 18S, ACTB, GAPDH, and HPRT. Primer sequences are listed in Table S5.

For the analysis of *S. aureus* gene expression, RNA from *in vivo* and *in vitro* samples was purified as described above in “RNA extraction from heart valve tissue samples for transcriptomic assays.” cDNA synthesis was carried out using the PrimeScript RT (Perfect Real Time) Reagent Kit (Takara, Ref. RR037A), following the manufacturer’s protocol. Quantitative PCR was then performed using Power SYBR Green PCR Master Mix (Applied Biosystems, Ref. 4367659) and the corresponding oligonucleotides (Table S5). Relative expression levels were calculated by normalizing the Ct values of target genes to the housekeeping gene *gyrB*.

### ELISA

Metalloproteinase (MMP)-3, -8, and -9 levels were quantified in valve protein extracts using ELISA kits following the manufacturer’s instructions (R&D Systems).

### Histology and immunohistochemistry

Porcine and human valves were fixed in 4% buffered formalin for 24 hours. Histological analyses were conducted on 5 µm thick sections stained with Movat pentachrome and Gram stain following the manufacturer’s instructions (Sigma). The double staining with alcian blue and sirius red was performed by immersing samples for 20 minutes in alcian blue (Sigma) and adding a 30-minute immersion in sirius red solution (1% in picric acid, Sigma). Immunohistochemical procedures were carried out using an automated immunostainer, Leica BOND-Polymer Refine Detection (Leica Biosystems, Madrid, Spain), in accordance with the manufacturer’s protocols for descriptive purposes. Primary antibodies and their respective working concentrations were as follows: CD31 (1:500), vimentin (1:1,000, Santa Cruz Biotechnology), CCL-2 (1:500), PAI-1 (1:1,000), TF (1:500), MMP-3 (1:500), MMP-9 (1:1000), CXCL-10 (1:1000), and CD68 (1:1,000). Poly-HRP anti-mouse or poly-HRP anti-rabbit IgG secondary antibodies were utilized as appropriate. Positive immunoreactive signals were developed using an enhanced 3,3′-diaminobenzidine (DAB) system (Leica Biosystems, Madrid, Spain). Subsequently, slides were counterstained with hematoxylin and mounted with DPX mounting medium (Merck/Sigma-Aldrich, Madrid, Spain).

Histological and immunohistochemistry preparations were imaged using bright-field microscopy with an automated imaging system (Nikon), as appropriate. Digital image analyses were performed for detailed histoanatomical characterization. Briefly, representative fields per section were imaged at 50 or 400× magnification, depending on the target and resolution requirements. Quantification of molecular targets was conducted using ImageJ software by applying binary thresholding to the red channel and calculating the percentage of tissue area positive for the signal. All measurements were normalized to the total tissue area analyzed. Image quantifications were independently performed by two blinded observers to ensure consistency and objectivity. Representative images are shown in the figures.

### Statistical analyses

Continuous data are presented as mean ± standard deviation (SD), while categorical data are expressed as frequency (percentage). Clinical data were analyzed using IBM SPSS Statistics 25.0.0.0 software. The normal distribution of data was assessed using the Kolmogorov-Smirnov test. One-way ANOVA was employed for continuous variables, and Pearson’s χ^2^ test was used for categorical variables to evaluate statistical differences between groups.

In pigs, molecular data were analyzed using GraphPad Software Inc., employing one-way ANOVA followed by Dunnett’s tests to assess differences between each etiological group and control valves. Student’s *t*-test was utilized to test differences between control and IE valves, and an unpaired *t* test to evaluate *S. aureus* gene expression levels in qRT-PCR experiments. The critical significance level for all analyses was set at a *P* value of <0.05.

## Data Availability

The raw RNA sequencing (RNAseq) FastQ files have been deposited in the NCBI under the BioProject number PRJNA1091634.

## SUPPLEMENTAL MATERIAL

Data set S1 mbio.02316-25-s0001.xlsx Differentially expressed genes in valve cells during *in vivo* endocarditis in pigs.

Data set S2 mbio.02316-25-s0002.xlsx Host functional pathways differentially expressed during endocarditis in the porcine model.

Data set S3 mbio.02316-25-s0003.xlsx Differential expression of *S. aureus* genes during *in vivo* endocarditis

Supplemental material mbio.02316-25-s0004.docx Supplemental figures, tables, and data set captions.

ASM does not own the copyrights to Supplemental Material that may be linked to, or accessed through, an article. The authors have granted ASM a non-exclusive, world-wide license to publish the Supplemental Material files. Please contact the corresponding author directly for reuse.
