# Erratum for Garcia et al., “Dual transcriptomic profiling of *Staphylococcus aureus* endocarditis in a porcine model reveals strong parallels with human infection”

**DOI:** 10.1128/mbio.03870-25

**Published:** 2026-01-13

**Authors:** Begoña García, Aritza Conty, Amaya Fernández-Celis, Daniel Mouzo, Carmen Gil, Nahiara Garmendia-Antoñana, Ana Navascues, Carmen Ezpeleta, Cristina Solano, Ivan Pasquier, Virginia Álvarez, Rafael Sádaba, David Gomez-Cabrero, Natalia López-Andres, Iñigo Lasa

## ERRATUM

Volume 16, no. 12, e02316-25, 2025, https://doi.org/10.1128/mbio.02316-25. Page 1: David Gomez-Cabrero’s surname should appear as given in this Erratum.

