# Supplementary material for: Dual transcriptomic profiling of Staphylococcus aureus endocarditis in a porcine model reveals strong parallels with human infection
Source: mBio. 2025 Oct 31;16(12):e02316-25. doi: 10.1128/mbio.02316-25 (PMC12691691; doi:10.1128/mbio.02316-25)
Supplement: Supplemental material — Supplemental figures, tables, and data set captions. [file mbio.02316-25-s0004.docx]

**SUPPLEMENTARY FIGURES**


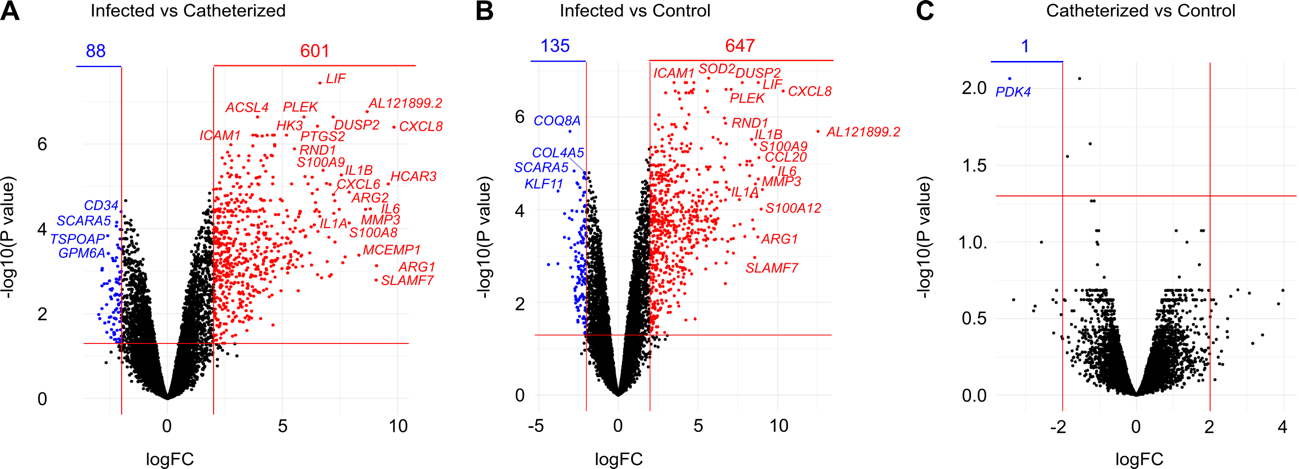


**Supplementary Figure S1. Analysis of differentially expressed genes in pig valvular tissues.** Volcano view of the comparisons between infected *vs.* catheterized (A), infected *vs.* control (B), and catheterized *vs.* control (C). Genes with differential expression with an adjusted p-value < 0.05 and log_2_ FC > 2/< -2 are shown (red and blue respectively). The number of up- and down-regulated genes is indicated. The most differentially regulated genes are labeled using the name of the orthologous gene in humans.


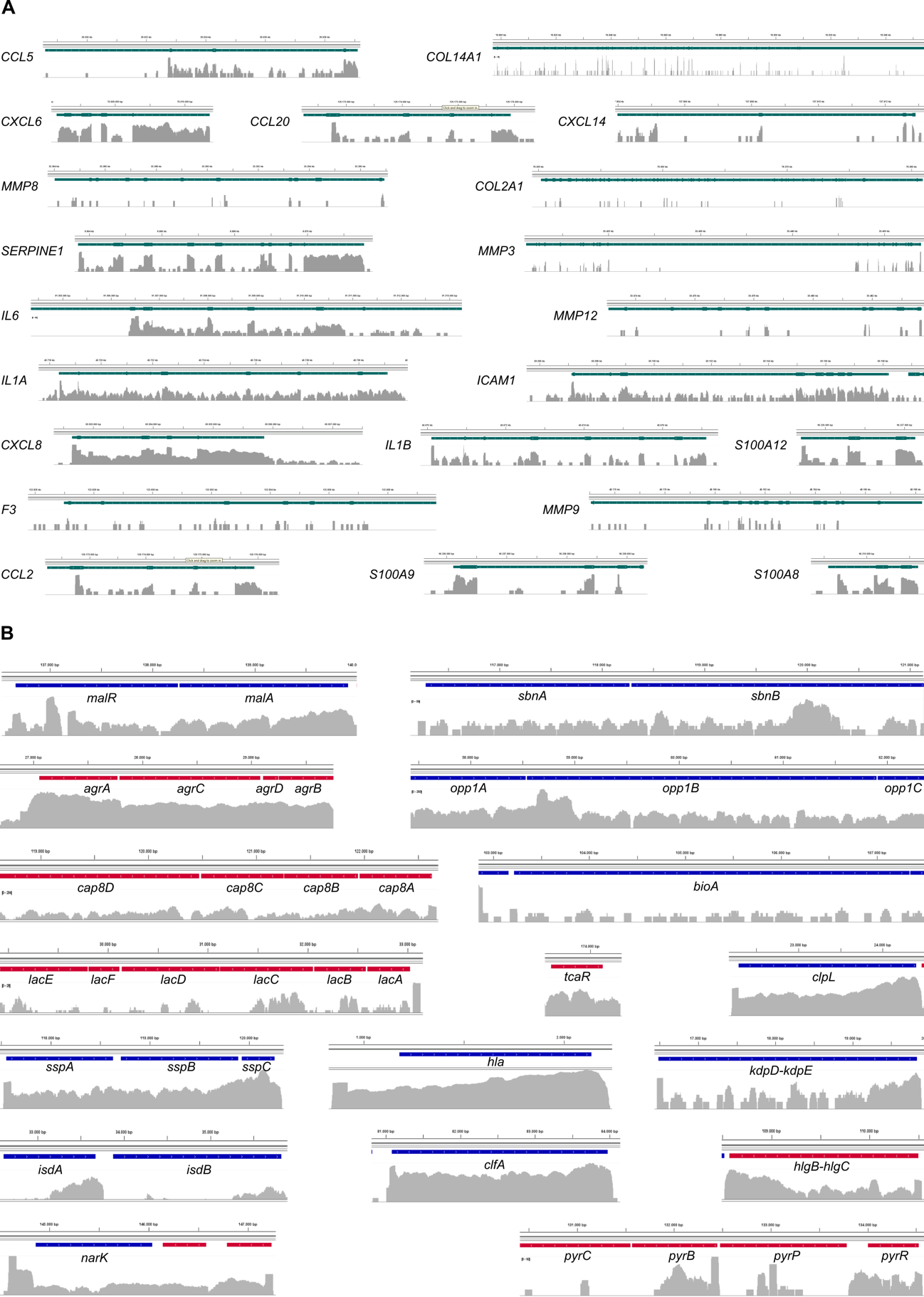


**Supplementary Figure S2. Dual RNAseq read coverage diagrams in the porcine IE model.** Read coverage is shown on a logarithmic scale for selected prominent genes from both the porcine host (A) and *S. aureus* (B). Gene names are labeled. Sample C51 was used to illustrate coverage. In panel B, *S. aureus* genes are color-coded blue or red based on the coding strand. However, due to inconsistent orientation of contigs in the reference genome, strand direction shown may not accurately reflect the true orientation of the genes in the *S. aureus* genome.


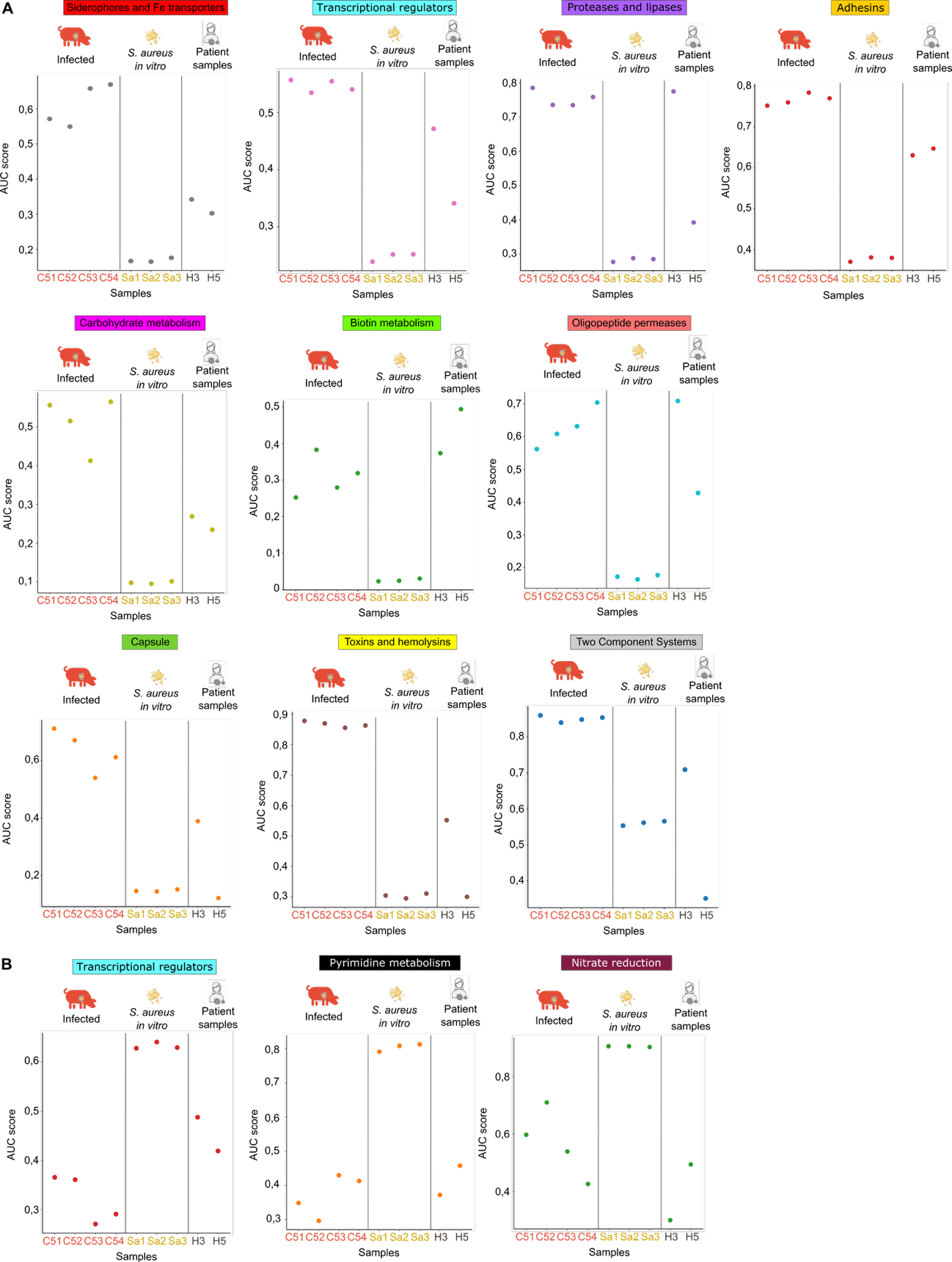


**Supplementary Figure S3. Activity scores of *S. aureus* differentially expressed functional pathways during *in vivo* human endocarditis calculated in each pig and patient sample.** *S. aureus* genes belonging to the differentially expressed functional pathways described in Fig. 9 were used to calculate the AUC ratio of each functional pathway for each of the pig and patient samples. (A) AUC ratios of the induced and (B) repressed set of genes. Functional pathways are indicated.

**SUPPLEMENTARY TABLES**

**Supplementary Table 1. Results of blood cultures performed throughout the experimental model of porcine endocarditis, symptoms on the day of euthanasia and results of *S. aureus* 16S rRNA PCRs from heart valves samples.** The result of the blood culture and PCR for *S. aureus* is indicated as positive (+) or negative (-). For blood cultures, the time to positivity is indicated, while for PCRs, the highest dilution with a positive result is reported.

| **Group** | **Animal number** | **Blood culture for *S. aureus*** | | | **Symptoms on Day 5** | | | | | **PCR 16S**  **from *S. aureus*** | |
| --- | --- | --- | --- | --- | --- | --- | --- | --- | --- | --- | --- |
|  |  | **Day 1** | **Day 3** | **Day 5** | **Fever** | | **Erythema** | **Mucus** | **Immovility** | **Aortic valve** | **Mitral valve** |
| Healthy | C60 |  |  | *-* | - | | - | - | - | - | - |
|  | C61* |  |  | - | - | | - | - | - | - | - |
|  | C62* |  |  | - | - | | - | - | - | - | - |
|  | C66 |  |  | - | - | | - | - | - | - | - |
|  | C67 |  |  | - | - | | - | - | - | - | - |
|  | C68* |  |  | - | - | | - | - | - | - | - |
| Catheter | C57 | *-* |  | *-* | - | | - | - | - | - | - |
|  | C58* | - |  | - | - | | - | - | - | - | - |
|  | C59* | - |  | *-* | - | | - | - | - | - | - |
|  | C63 | *-* |  | *-* | - | | - | - | - | Failed | - |
|  | C64 | - |  | *-* | - | | - | - | - | - | - |
|  | C65* | - |  | - | - | | - | - | - | - | Failed |
| Infected | C51* | - | - | + (9 h 33 min) | + | | + | + | + | + (Dil 1/200) | + (Dil 1/30) |
|  | C52* | - | - | + (11 h 23 min) | + | | + | + | + | + (Dil 1/50) | + (Dil 1/30) |
|  | C53* | *-* | - | + (10 h 03 min) | + | | + | + | + | + (Dil 1/100) | - |
|  | C54* | - | - | + (9 h 19 min) | + | | + | + | + | + (Dil 1/50) | + (Dil 1/50) |
|  | C55 | - | Dead | | | | | | | | |
|  | C56 | - | *-* | + (9 h 19 min) | + | + | | + | + | + (Dil 1/2) | + ( Dil 1/5) |

*Animals used for RNA dual seq assay

**Supplementary Table 2. Characteristics of total RNA extracted from heart valves in the porcine endocarditis model.** The table presents the total RNA yield, RNA integrity as assessed by the RIN (RNA Integrity Number), and capillary electrophoresis profiles showing rRNA peaks in each sample. RIN values were determined using the eukaryotic analysis mode on the Bioanalyzer. In samples from the infected group, RIN values were not determined (ND) due to the presence of both porcine (18S/28S) and *S. aureus* (16S/23S) rRNA. Despite this, electropherogram traces indicated that RNA quality was acceptable. Bacterial rRNA peaks in the infected samples were identified by comparison with RNA profiles obtained from purified *S. aureus* cultures.


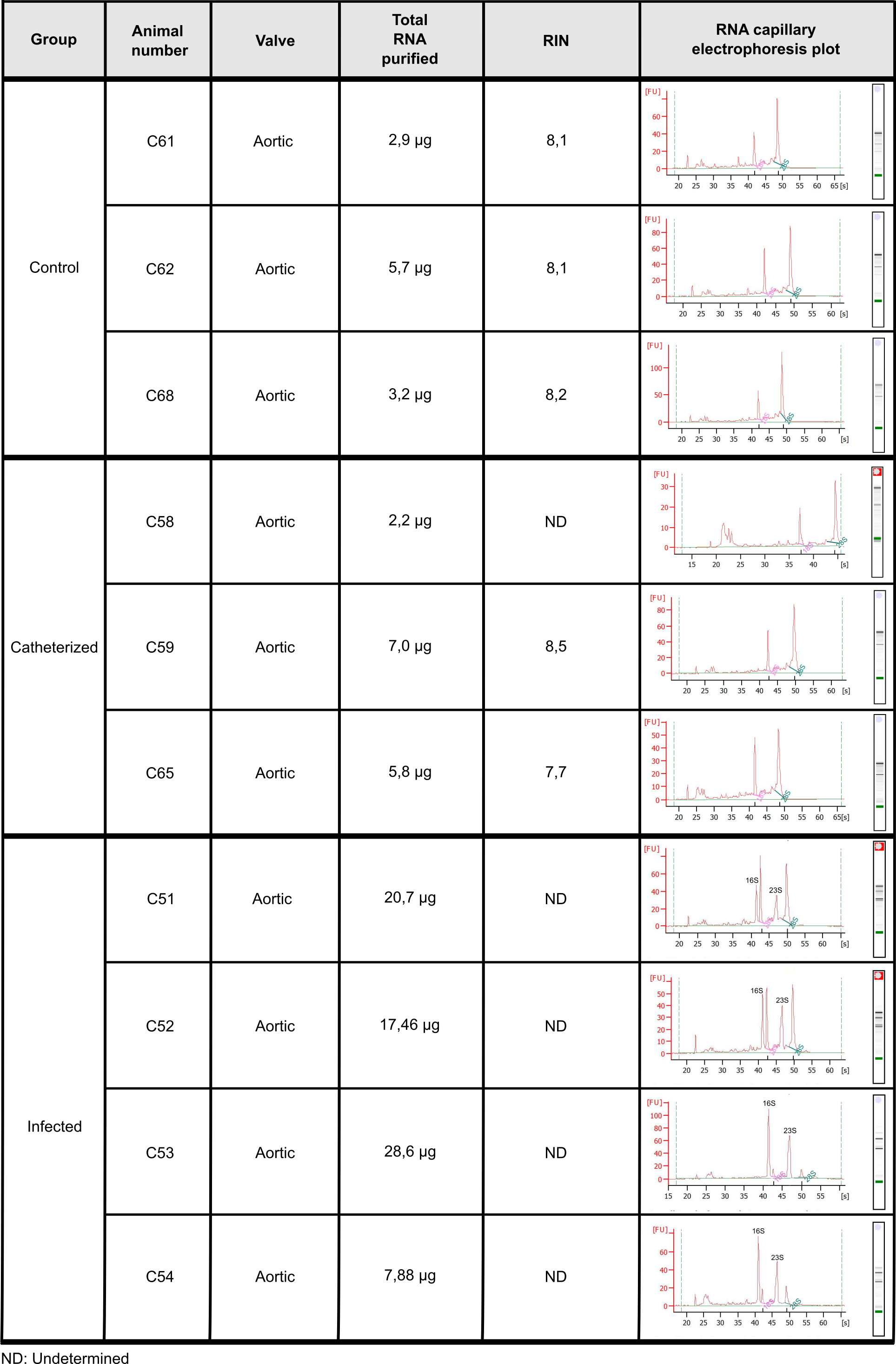


**Supplementary Table 3. Summary of read mapping statistics of the transcriptomic assays**. For each sample analyzed, the total number of reads and the percentage of total reads mapped to the host and the bacteria are shown, calculated relative to the total number of reads in each sample. The percentages of reads uniquely aligned and reads aligned to different transcript classes are also shown. These percentages refer to the total number of reads that map to the host or the bacteria, respectively. mRNA: messenger RNA, Mt_rRNA: mitochondrial ribosomal RNA, Mt_tRNA: mitochondrial transfer RNA, rRNA: ribosomal RNA, scaRNA: small cajal body-specific RNA, snoRNA: small nucleolar RNA, snRNA: small nuclear RNA, CDS: Coding Sequence, ncRNA: non-coding RNA, tRNA: transfer RNA.

| **Group** | **Animal number** | **Valve** | **Total sequenced reads** | **% of host reads** | | | | | | | | | **% of bacterial reads** | | | | |
| --- | --- | --- | --- | --- | --- | --- | --- | --- | --- | --- | --- | --- | --- | --- | --- | --- | --- |
|  |  |  |  | **Aligned reads** | **Uniquely aligned reads** | mRNA | Mt-rRNA | Mt-tRNA | rRNA | scaRNA | snoRNA | snRNA | **Aligned reads** | CDS | ncRNA | rRNA | tRNA |
| Control | C61 | Aortic | 29,876,160 | 99.43 | 38.70 | 2.34 | 0.14 | 0.001 | 0.060 | 0.71 | 6.83 | 0.79 | - | - | - | - | - |
|  | C62 | Aortic | 33,003,684 | 99.34 | 40.74 | 4.40 | 0.19 | 0.002 | 0.035 | 0.77 | 4.98 | 0.59 | - | - | - | - | - |
|  | C68 | Aortic | 30,666,811 | 99.51 | 42.59 | 3.23 | 0.19 | 0.002 | 0.038 | 0.92 | 7.56 | 0.53 | - | - | - | - | - |
| Catheterized | C58 | Aortic | 35,056,161 | 98.97 | 33.70 | 2.47 | 0.11 | 0.002 | 0.142 | 0.48 | 6.42 | 0.81 | - | - | - | - | - |
|  | C59 | Aortic | 32,571,262 | 99.58 | 34.63 | 2.12 | 0.18 | 0.002 | 0.025 | 0.62 | 5.37 | 0.87 | - | - | - | - | - |
|  | C65 | Aortic | 29,978,973 | 99.39 | 39.11 | 3.72 | 0.23 | 0.002 | 0.048 | 0.83 | 6.15 | 0.62 | - | - | - | - | - |
| Infected | C51 | Aortic | 44,909,599 | 63.92 | 26.87 | 3.27 | 0.50 | 0.009 | 0.032 | 0.73 | 5.50 | 0.80 | 35.54 | 4.83 | 0.34 | 42.10 | 0.15 |
|  | C52 | Aortic | 41,368,565 | 58.78 | 21.82 | 3.60 | 0.47 | 0.010 | 0.024 | 0.70 | 4.85 | 0.82 | 40.51 | 6.09 | 0.42 | 44.87 | 0.23 |
|  | C53 | Aortic | 31,377,935 | 13.08 | 4.32 | 3.68 | 0.73 | 0.005 | 0.033 | 0.73 | 4.69 | 0.77 | 86.43 | 2.28 | 0.10 | 54.30 | 0.17 |
|  | C54 | Aortic | 27,292,217 | 27.51 | 9.71 | 4.16 | 0.66 | 0.011 | 0.048 | 0.45 | 3.52 | 0.88 | 71.76 | 7.78 | 0.22 | 42.24 | 0.56 |
| *S. aureus*  *in vitro* | Sa1 | - | 10,372,574 | - | - | - | - | - | - | - | - | - | 96.47 | 46.03 | 2.93 | 7.22 | 1.09 |
|  | Sa2 | - | 10,365,139 | - | - | - | - | - | - | - | - | - | 96.52 | 43.52 | 2.95 | 7.12 | 1.02 |
|  | Sa3 | - | 10,769,103 | - | - | - | - | - | - | - | - | - | 96.29 | 47.55 | 2.64 | 5.69 | 1.00 |
| Patient samples | H1* | Mitral | 36,254,322 | 99.17 | 39.92 | 3.41 | 0.57 | 0.014 | 0.002 | 0.72 | 3.28 | 2.56 | 0.04 | 1.63 | 0.04 | 0.85 | 0.00 |
|  | H2 | Mitral | 32,448,768 | 97.97 | 38.35 | 2.66 | 0.24 | 0.039 | 0.002 | 0.32 | 1.38 | 3.92 | 0.06 | 5.10 | 0.37 | 3.21 | 0.25 |
|  | H3 | Mitral | 31,460,382 | 83.22 | 35.82 | 4.09 | 0.20 | 0.028 | 0.002 | 0.31 | 1.79 | 5.22 | 15.15 | 35.22 | 0.82 | 2.01 | 0.46 |
|  | H4 | Aortic | 15,750,958 | 95.74 | 43.57 | 6.15 | 0.30 | 0.068 | 0.004 | 0.12 | 2.06 | 2.70 | 0.19 | 21.50 | 0.18 | 31.93 | 1.80 |
|  | H5* | Mitral | 19,963,999 | 60.00 | 33.27 | 7.08 | 0.28 | 0.363 | 0.003 | 0.08 | 0.67 | 2.53 | 35.75 | 10.18 | 0.14 | 40.54 | 1.55 |
|  | H6 | Mitral | 18,355,048 | 97.68 | 38.58 | 4.87 | 0.87 | 0.195 | 0.005 | 0.16 | 1.78 | 4.93 | 0.23 | 5.54 | 0.10 | 25.26 | 5.26 |
|  | H7 | Mitral | 21,016,537 | 95.21 | 53.13 | 8.41 | 0.83 | 0.245 | 0.005 | 0.11 | 1.56 | 3.39 | 0.16 | 18.52 | 0.27 | 29.47 | 1.29 |

* Independent samples from the same patient.

**Supplementary Table 4. Characteristics of the RNA extracted from heart valves of patients with *S. aureus* endocarditis.** The table displays the total RNA yield, RNA integrity as assessed by the RIN (RNA Integrity Number), and capillary electrophoresis profiles showing rRNA peaks in the samples. RIN values were determined using the eukaryotic mode on the Bioanalyzer. In one sample, in addition to the characteristic peaks for human rRNA (18S/28S), peaks corresponding to *S. aureus* rRNA (16S/23S) were also detected. Identification of the bacterial rRNA peaks was confirmed by comparison with RNA profiles from purified *S. aureus* cultures.

**
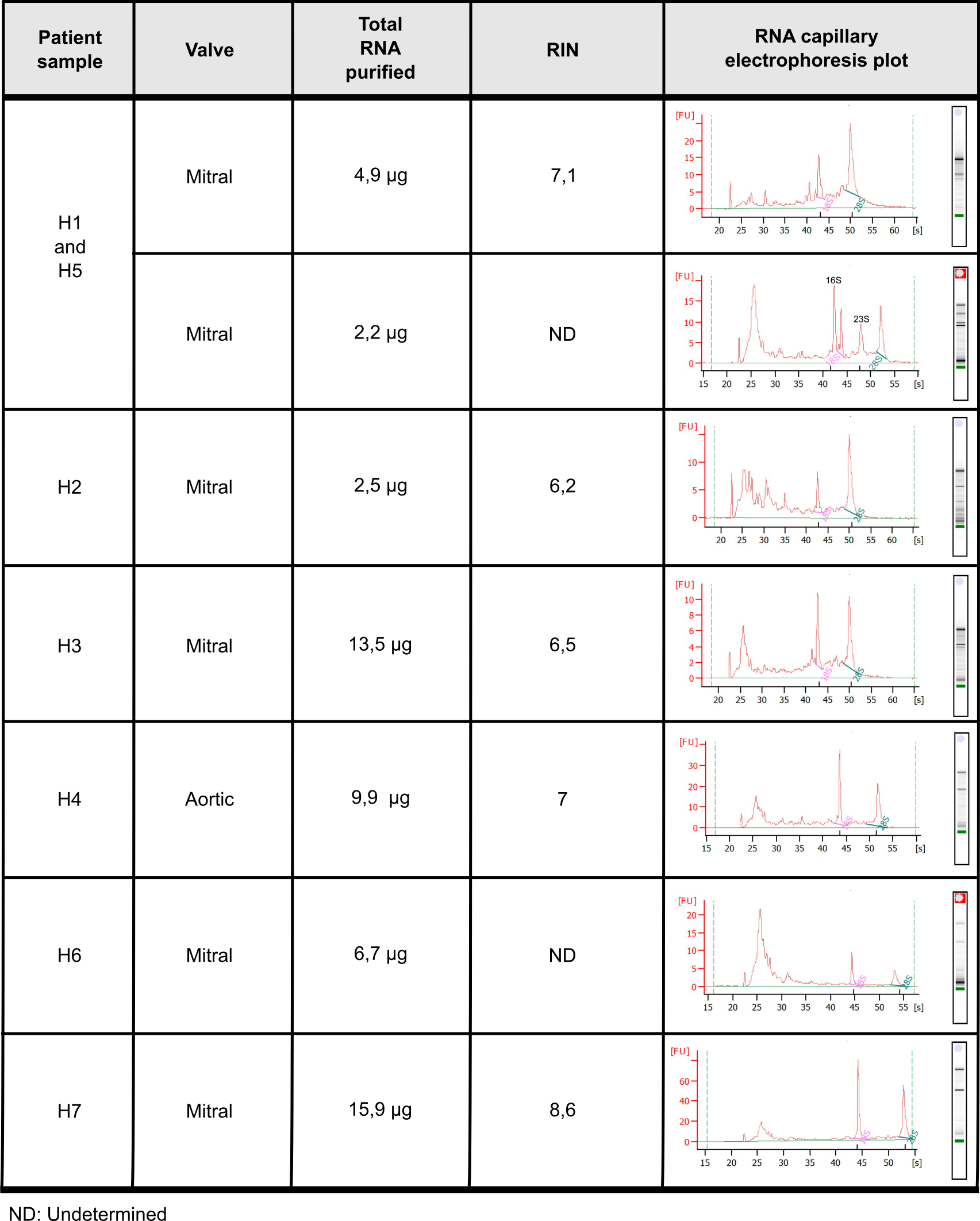
**

**Supplementary Table 5. Primers used in real time PCR analysis.**

| **Primers used in pig samples** | | |
| --- | --- | --- |
| **Gene** | **Primer** | **Sequence (5´to 3´)** |
| 18S | Forward  Reverse | GAACGCCACTTGTCCCTCTA  GACTCAACACGGGAAACCTC |
| PECAM1 | Forward  Reverse | CACCGAGGTCTGGGAACAAA  GGAGCCTTCCGTTCTAGAATATC |
| CDH5 | Forward  Reverse | CGAGAGACAGTGCCCATGTA  AATGTGTACCTGGTCTGGGTG |
| VWF | Forward  Reverse | CAGGGGGACCAAAGCATCTC  TTGCCGCTCCCATCAATTCT |
| VIM | Forward  Reverse | CGCCAGCAGTATGAGAGTGT  AGCAGGTCTTGGTATTCACGA |
| ACTA2 | Forward  Reverse | CAGCCAGGATGTGTGAAGAA  TCACCCCCTGATGTCTAGGA |
| CCL2 | Forward  Reverse | TCCCACACCGAAGCTTGAATC  TGGAGAATTAATTGCATCTGGCTG |
| CCL3 | Forward  Reverse | ATGGCTCTCTGCAACCAGTTCT  GGCTTCGCTTGGTTAGGAAGATGA |
| CCL5 | Forward  Reverse | CATCAGCCTCCCCATATGCCTC  CACACACCTGGCGGTTCTTT |
| CCL20 | Forward  Reverse | CTGCTCTACCTCTGCAGCAA  TCTTCTTGACTCTTTGACTGAGG |
| S100A8 | Forward  Reverse | CTGACGGATCTGGAGAGTGC  GGTTTCTGCGTCCTTTTTCTTCA |
| S100A9 | Forward  Reverse | CCTGTGAAGCAATCTTCCCG  CATCTTCCTGCACTCCAAGCC |
| S100A12 | Forward  Reverse | CCCAGCTTTGTAGGGGAGAAG  CCTGCTGATCGCTGTTCTCA |
| IL-1 α | Forward  Reverse | CCAGCCAGCACAGAAGTGAA  TCCCCAGGAAGTGGCTCATA |
| IL-1β | Forward  Reverse | ACCTGGACCTTGGTTCTCTG  CATCTGCCTGATGCTCTTGT |
| IL-6 | Forward  Reverse | AATGTCGAGGCTGTGCAGATT  TGGTGGCTTTGTCTGGATTC |
| ICAM-1 | Forward  Reverse | AAAGGAGGCTCCATGAAGGT  TGCCATCGTTTTCCACATTA |
| F3 | Forward  Reverse | TAGTCAGGGTGAACGGCAC  GGTCGTGGCCTTTTTCTTTCC |
| SERPINE-1 | Forward  Reverse | CCTGGTTCTGCCCAAGTTCTC  GATCTTCACCTTTTGCAGGGC |
| MMP3 | Forward  Reverse | CCCGTTGAGCCCACAGAAT  TTGCGCCAAAAATGTCTGTCC |
| MMP8 | Forward  Reverse | GCCACACTCCGTGGAGAAAT  GCCCAGTACTTGTTGCCTTTG |
| MMP9 | Forward  Reverse | AGAACTTCTGGAGGTTCGACG  ACCTGATTCACCTCGTTCCG |
| MMP12 | Forward  Reverse | ATCCTGGTTCTGCAGGTCAC  ATGGAGTGCCCATTGACTTT |
| CXCL6 | Forward  Reverse | ACGCTGAGAGTAAACCCCAA  TTCTTCAGGGAGGCTACCAC |
| CXCL8 | Forward  Reverse | GAAGTTTTTGAAGAGGGCTGAGA  TGCTTGAAGTTTCACTGGCATC |
| CXCL14 | Forward  Reverse | GGACCCAAGATCCGCTACAG  ACGCTCTTGGTGGTGATGATAA |
| COL2A1 | Forward  Reverse | GAAGGATGGCTGCACGAAAC  TGTCAATGATGGGGAGGCG |
| COL14A1 | Forward  Reverse | ACCCCAGAATAGAATGGCACT  AATGTAGTTCAAAGCAAGACCTGT |
| GADPH | Forward  Reverse | ACCCAGAAGACTGTGGATGG  ACGCCTGCTTCACCACCTTC |
| HPRT | Forward  Reverse | AACCTTGCTTTCCTTGGTCA  TCAAGGGCATAGCCTACCAC |
| ACTB | Forward  Reverse | TCCCTGGAGAAGAGCTACGA  TGTTGGCGTAGAGGTCCTTC |
| **Primers used in human samples** | | |
| 18S | Forward  Reverse | CGCCGCTAGAGGTGAAATTC  TCTTGGCAAATGCTTTCGC |
| PECAM1 | Forward  Reverse | TTCCCACGCCAAAATGTTA  CACAGCACATTGCAGCACA |
| CDH5 | Forward  Reverse | CAGCCCAAAGTGTGTGAGAA  CGGTCAAACTGCCCATACTT |
| VWF | Forward  Reverse | TAAGTCTGAAGTAGAGGTGG  AGAGCAGCAGGAGCACTGGT |
| VIM | Forward  Reverse | CCTTGAACGCAAAGTGGAAT  TTGGCAGCCACACTTTCATA |
| ACTA2 | Forward  Reverse | ACTGCCTTGGTGTGTGACAATGG  TGGTGCCAGATCTTTTCCATG |
| CCL2 | Forward  Reverse | TCCCAAAGAAGCTGTGATCTTCA  TTTGCTTGTCCAGGTGGTCC |
| CCL3 | Forward  Reverse | ATGGCTCTCTGCAACCAGTTCT  GGCTTCGCTTGGTTAGGAAGATGA |
| CCL5 | Forward  Reverse | CTGCTGCTTTGCCTACATTG  TGTACTCCCGAACCCATTTC |
| CCL20 | Forward  Reverse | GGCGAATCAGAAGCAAGCAA  GGATTTGCGCACACAGACAA |
| S100A8 | Forward  Reverse | AAGGGGAATTTCCATGCCGT  CGTCTGCACCCTTTTTCCTG |
| S100A9 | Forward  Reverse | TCCTCGGCTTTGACAGAGTG  TGCCCCAGCTTCACAGAGTA |
| S100A12 | Forward  Reverse | CATTCCTGTGCATTGAGGGGTTA  GGTGTCAAAATGCCCCTTCC |
| IL-1α | Forward  Reverse | CGTTTGAGTCAGCAAAGAAGTCAAG  GAGTGGGCCATAGCTTACATGA |
| IL-1β | Forward  Reverse | AGCTGATGGCCCTAAACAGA  GCATCTTCCTCAGCTTGTCC |
| IL-6 | Forward  Reverse | AGTTCCTGCAGAAAAAGGCAAAG  CATTTGCCGAAGAGCCCTCA |
| ICAM-1 | Forward  Reverse | TGATGGGCAGTCAACAGCTA  CTCCCGTTTCAGCTCCTTCT |
| F3 | Forward  Reverse | GTACTTGGCACGGGTCTTCT  GGCTGTCCGAGGTTTGTCTC |
| SERPINE-1 | Forward  Reverse | CGCTGTCAAGAAGACCCACA  CTCAGAGGTGCCTTGCGATT |
| CXCL6 | Forward  Reverse | CCAAGGCAGAAGTGATAGCCA  ATCAGAGGAGCTTTTGGGTCC |
| CXCL8 | Forward  Reverse | TGGACCCCAAGGAAAAGTGG  TGTTGTTGCTTCTCAGTTCTCT |
| CXCL14 | Forward  Reverse | TGAGAAGCGCAGGGTCTATG  ACCCTCGGTAAAAGTGCTGG |
| COL2A1 | Forward  Reverse | GAAGGATGGCTGCACGAAAC  TGTCAATGATGGGGAGGCG |
| COL8A2 | Forward  Reverse | CGACCTGAAAGCACGTCCA  AGAGGCATTTCAGTAGCAGCA |
| COL14A1 | Forward  Reverse | ACCCCAGAATAGAATGGCACT  AATGTAGTTCAAAGCAAGACCTGT |
| GADPH | Forward  Reverse | ACCAGCCCCAGCAAGAGCACAAG TTCAAGGGGTCTACATGGCAACTG |
| HPRT | Forward  Reverse | TTGCTTTCCTTGGTCAGGCA  ATCCAACACTTCGTGGGGTC |
| ACTB | Forward  Reverse | GCCGCCAGCTCACCAT TCGATGGGGTACTTCAGGGT |
| **Primers used in *S. aureus* samples** | | |
| *hla* | Forward  Reverse | GGGACCATATGACAGAGATTCTTG  GCAAAGTCTGGTGAGAATCCTG |
| *hlgB* | Forward  Reverse | CGAGATAGCTTCCACCCAAC  TGTTGCGCTATGAAGTTTTGG |
| *isdB* | Forward  Reverse | ACCCTGCAATAAAAGACAAAGATC  ACTGTTGAGTGCCATCTTTCT |
| *cap8C* | Forward  Reverse | TGGTGATGTGCGTGATAGTC  TGTCTTCACTGCCTCAACTG |
| *lacC* | Forward  Reverse | TGAACGATGCCAAAACAAAGG  TCCAATACTGTTTGTAAAGTTGCAC |
| *clpL* | Forward  Reverse | GTAGCTTCCTATTTGTCGGTCC  ATCAAGTCGAATCAGTGCCTC |
| *sspB* | Forward  Reverse | TGCGTACATTATACCCTGAAGTAAG  TCTCTACCTTGTGATTTACCGTATTC |
| *opp1B* | Forward  Reverse | GCGCTCATGTTTCCATTGATG  CGTTTCTGCAATCAACTCTGG |
| *agrA* | Forward  Reverse | CGAAGACGATCCAAAACAAAGAG  GCTCAAGCACCTCATAAGGAT |
| *kdpD* | Forward  Reverse | TTTACGTATGCCCACCTAAGC  ATACTCCCACTAATTGCCACAG |
| *clfA* | Forward  Reverse | GTTGACGAATGTGACAGTTGG  CACCTTGAACAGCCTCATTTG |
| *bioA* | Forward  Reverse | CACTTGAGAAACAGTTACATGCG  CAACTCCAAACATTAAGCCTCG |
| *malR* | Forward  Reverse | TGACAGCAACGTTCAATGATTC  TGACCTAACATTCGAGGCTTG |
| *tcaR* | Forward  Reverse | AAATCACGCAAAGACAAGGTG  TGCTCTACCTTTGTCAGTTAAGG |
| *pyrP* | Forward  Reverse | CAAATTAAGTGGGACAGCATGG  CTCCAGGATTTTCATACATTGCC |
| *narK* | Forward  Reverse | GGAATTGACAAAGTAGATGCTGG  CCTATTGGTCTCAAGAATGTTGC |
| *gyrB* | Forward  Reverse | TCAGCGTTAGATGTAGCAAGTC  ACGACCAGATTTTGTAGACCC |

**SUPPLEMENTARY DATASETS**

**Supplementary dataset 1. Differentially expressed genes in valve cells during *in vivo* endocarditis in pigs.** Differentially expressed genes retrieved when RNA-seq data from valve samples of the infected group *vs* control group comparison and the infected group *vs* catheterized group comparison were analyzed (p value adjust < 0.05 and log_2_ FC > 2 / < -2). First tab: upregulated genes, second tab: downregulated genes.

**Supplementary dataset 2. Host functional pathways differentially expressed during endocarditis in the porcine model.** This dataset includes Gene Ontology (GO) pathways significantly enriched in the infected group compared to both the control and catheterized groups (adjusted p < 0.05). No significantly enriched pathways were identified in the catheterized *vs.* control group comparison.

**Supplementary dataset 3. Differential expression of *S. aureus* genes during *in vivo* endocarditis.** This dataset presents *S. aureus* genes that were differentially expressed in infected pig valve samples relative to *S. aureus* S54F9 cultured *in vitro* (adjusted p < 0.05; log₂ fold change > 2 or < -2). The first tab lists upregulated genes; the second tab lists downregulated genes. Genes are color-coded by functional category: siderophores and iron transporters (red), hemolysins (yellow), two-component systems (grey), capsule (dark green), biotin metabolism (light green), regulators (light blue), proteases (purple), adhesins (orange), carbohydrate metabolism (pink), oligopeptide permeases (light pink), nitrate reduction (garnet), pyrimidine metabolism (black), and hypothetical proteins (dark blue).
